# Tembusu virus neuroinvasion and neuropathogenesis: lessons from blood–brain barrier dysfunction in neurotropic flaviviruses

**DOI:** 10.3389/fvets.2026.1939599

**Published:** 2026-09-23

**Authors:** Zheng Ni, Zicheng Zhang, Ting Zhou, Yinchu Zhu, Suxin Huo, Jionggang Hua, Xuejun Wu, Tao Yun, Liu Chen, Weicheng Ye, Yuan Fu, Zhuanli Wu, Guijun Wang, Zhuangli Bi

**Affiliations:** 1 Institute of Animal Husbandry and Veterinary Science, Zhejiang Academy of Agricultural Sciences, Hangzhou, China; 2 College of Animal Science and Technology, Anhui Agricultural University, Hefei, China; 3 College of Veterinary Medicine, Nanjing Agricultural University, Nanjing, China; 4 Zhejiang Center for Animal Disease Prevention and Control, Hangzhou, China

**Keywords:** blood–brain barrier dysfunction, CNS disease, flavivirus, neuroinflammation, neuroinvasion, neuropathogenesis, Tembusu virus

## Abstract

Central nervous system (CNS) pathogenesis remains a critical but incompletely resolved aspect of flavivirus infection. Neurotropic flaviviruses usually initiate replication in peripheral tissues but can subsequently access the CNS, causing encephalitis, neurological dysfunction, or developmental abnormalities. Because these events occur along a continuum from peripheral infection to CNS injury, their temporal and mechanistic relationships are often difficult to define. In many studies, brain viral detection, blood–brain barrier (BBB) dysfunction, neuroinflammation, and neurological signs are reported together, although they represent different steps in disease progression. Studies of Japanese encephalitis virus, West Nile virus, Zika virus, dengue virus, and tick-borne encephalitis virus show that BBB dysfunction is an important component of flavivirus-associated neurological disease, but no single conserved mechanism applies across flaviviruses. Endothelial activation, cytokine and chemokine signaling, tight junction remodeling, matrix metalloproteinase activity, leukocyte trafficking, glial inflammatory amplification, and viral genetic variation can each shape barrier-related phenotypes, with distinct timing and relative importance depending on virus species and experimental context. Tembusu virus (TMUV) is an evolving avian flavivirus responsible for widespread waterfowl outbreaks, with ongoing genetic diversification and an expanding recognized host and vector range. Increasing evidence of neurological signs, brain lesions, and viral detection in neural tissues indicates that CNS-associated pathogenicity is an important feature of TMUV infection. However, the routes of TMUV CNS entry, the temporal relationship between BBB dysfunction and early brain infection, the viral genetic basis of strain-dependent neurological phenotypes, and the contribution of host inflammation to BBB injury remain unclear. Here, we review current advances in flavivirus-associated BBB dysfunction and use comparative flavivirus evidence to guide a stage-specific interpretation of TMUV neuroinvasion and neuropathogenesis. We argue that TMUV-associated neurological disease should be interpreted along a peripheral-to-CNS sequence that separates CNS access, BBB dysfunction, post-entry brain replication, neuroinflammatory amplification, and clinical disease. By linking CNS-associated phenotypes to defined disease steps, this approach may help explain the emergence of TMUV strains with enhanced neurological pathogenicity and provide a clearer basis for evaluating attenuation and vaccine safety.

## 1 Introduction

Flaviviruses comprise a diverse group of arthropod-borne, positive-sense RNA viruses, several of which are capable of invading the central nervous system (CNS) and causing encephalitis, meningitis, neurodevelopmental abnormalities, or other neurological manifestations (1, 2). Although CNS disease represents only one facet of flavivirus pathogenesis, it is often associated with severe clinical outcomes and long-term neurological sequelae (3). A central issue in flavivirus neuropathogenesis is how and when viruses that initially replicate in peripheral tissues overcome or bypass the specialized barriers that normally protect the CNS. Among these barriers, the blood–brain barrier (BBB) is particularly important because it not only restricts hematogenous viral entry into the CNS, but also provides a dynamic neurovascular interface through which systemic inflammation, endothelial activation, immune-cell trafficking, and local CNS infection can converge to promote barrier dysfunction and neurological injury (4, 5).

Accumulating evidence from well-studied neurotropic flaviviruses, including Japanese encephalitis virus, West Nile virus, Zika virus, tick-borne encephalitis virus, and dengue virus, indicates that BBB dysfunction does not follow a single conserved pattern. Rather, flavivirus-associated BBB dysfunction involves several overlapping processes, including endothelial activation, cytokine and chemokine signaling, tight junction remodeling, matrix metalloproteinase activity, leukocyte trafficking, and glial inflammatory amplification. Their relative importance varies across viral systems, host contexts, and stages of infection (6–8). These mechanisms provide a useful mechanistic context for understanding less extensively studied flaviviruses with emerging CNS-associated pathogenicity.

Tembusu virus (TMUV) is an avian flavivirus that has caused major disease outbreaks in waterfowl and has continued to diversify genetically, with broader host and vector involvement increasingly recognized (9, 10). This diversification has been associated with differences in host distribution, tissue involvement, and pathogenic phenotype among TMUV lineages and strains. These differences have shifted attention beyond classical reproductive and systemic disease toward CNS-associated pathogenicity. Neurological signs, CNS lesions, and viral detection in brain tissues have now been reported in several TMUV infection settings, indicating that CNS involvement is no longer a incidental observation in TMUV pathogenesis (11, 12). These observations highlight the need to move from documenting CNS involvement to defining the route of CNS entry, the involvement of CNS barriers, and the mechanisms by which viral replication and host inflammation produce neurological injury. This review examines BBB dysfunction in neurotropic flavivirus infection and uses comparative flavivirus evidence to guide a stage-specific interpretation of TMUV neuroinvasion and neuropathogenesis.

### 1.1 Neurotropic flaviviruses and CNS disease

Flaviviruses differ markedly in their ability to reach, infect, and damage the CNS. JEV and WNV represent the classical encephalitic pattern, in which peripheral infection can progress to CNS invasion, viral encephalitis, neuronal injury, and long-term neurological sequelae (13, 14). TBEV also causes CNS disease, but its clinical spectrum is often framed around meningitis, meningoencephalitis, and neurological dysfunction in endemic regions (15). ZIKV illustrates a different form of neurotropism, with disease linked to infection of neural progenitor cells and congenital neurodevelopmental abnormalities, in addition to neurological complications in adults (16). DENV is less commonly classified as a prototypical neurotropic flavivirus, yet CNS involvement and neurological manifestations are increasingly reported in severe or atypical dengue cases (17). These differences are important because BBB dysfunction may contribute to CNS disease in different ways depending on the virus, host context, and stage of infection.

Flavivirus-associated CNS disease should be interpreted by separating CNS access from post-entry events. After peripheral infection, a virus may reach CNS-associated tissues, infect susceptible neural or barrier-associated cells, replicate locally, and trigger neuroinflammation or tissue injury (18). These steps correspond to related but distinct concepts: neuroinvasion refers to CNS access after peripheral infection, neurotropism to infection of neural or CNS-associated cells, neurovirulence to disease severity after CNS access, and neuropathogenesis to the broader process leading to neurological injury. Direct CNS inoculation tests replication or disease potential after the entry barrier has been bypassed, not neuroinvasion. Likewise, CNS access does not necessarily lead to severe disease if local replication or inflammatory amplification is limited.

A key question is when and how the virus gains access to the CNS. Current flavivirus research therefore focuses not only on whether virus replicates in the brain, but also on which anatomical routes are involved and how barrier dysfunction relates to CNS infection and neuroinflammatory injury. These questions are particularly relevant for emerging or less extensively characterized flaviviruses, including TMUV, in which neurological manifestations have been reported but the sequence from peripheral infection to neuropathology remains incompletely defined.

### 1.2 Tembusu virus evolution and emerging CNS-associated pathogenicity

TMUV is a mosquito-borne flavivirus within the Ntaya virus group and has become an important avian pathogen. In mainland China, it drew major attention after large-scale outbreaks in egg-laying ducks beginning in 2010, when the most prominent field presentation was a sharp decline in egg production together with reproductive and systemic disease manifestations (19, 20). Subsequent surveillance and phylogenetic studies showed that TMUV did not remain a single outbreak-associated lineage. Instead, it continued to circulate and diversify into multiple clusters and subclusters, including Cluster 2 lineages such as Cluster 2.1, Cluster 2.2, and Cluster 2.3, as well as more recently recognized Cluster 3 viruses (9, 11, 12, 21). Early epidemic strains in China were mainly linked to Cluster 2, especially Cluster 2.2, whereas later studies identified additional lineages with different host distributions and pathogenic features (22). In parallel, TMUV has been detected or isolated from ducks, geese, chickens, sparrows, and mosquitoes, indicating broader host and vector involvement than was appreciated during the initial duck-associated outbreaks (10, 23–26). The strength of evidence, however, differs across host and vector contexts. Ducks and geese remain the best-established waterfowl hosts with clear disease associations, whereas chicken-origin Cluster 3.2 viruses have shown infectivity and pathogenicity in chicks and ducklings. House sparrow isolates support wild-bird infection, and mosquito-derived viruses, together with persistence in *Culex tritaeniorhynchus*, support vector-associated circulation. Thus, TMUV involves a broader host–vector ecology than initially recognized, but not all detected species represent the same level of host association or adaptation.

Subsequent studies have increasingly reported CNS involvement during infection with specific TMUV lineages and in particular host species, including neurological signs, brain lesions, viral detection in brain tissues, and nonsuppurative encephalitis (27). Importantly, several variant or non-classical TMUV lineages appear to show stronger CNS-related pathogenicity than early duck-associated epidemic strains. For example, a gosling-origin TMUV strain belonging to Cluster 2.1 caused severe neurological dysfunction in experimentally infected goslings and showed high viral loads in brain tissues, suggesting enhanced CNS-associated pathogenicity in this lineage (11). More recently, chicken-origin Cluster 3.2 TMUV was shown to infect chicks and ducklings and induce tissue lesions including brain lesions (26), and a goose-origin Cluster 3 strain caused severe neurological signs in goslings and displayed pathogenicity in ICR mice (12). These findings suggest that TMUV evolution and lineage diversification may be linked not only to broader host and vector involvement, but also to differences in CNS involvement, tissue distribution, CNS access, post-entry brain replication, and host inflammatory responses.

Despite these observations, the pathogenic sequence leading to TMUV-associated CNS disease remains unresolved. CNS involvement may arise at several points in the infection course, including peripheral viral burden, CNS barrier access, local cell susceptibility, brain replication, and inflammatory injury. These possibilities have different biological implications: a strain with higher viremia may increase viral exposure at CNS barriers, whereas a strain with stronger brain replication may worsen disease only after CNS entry has occurred. Distinguishing these possibilities is essential for interpreting TMUV CNS-associated phenotypes. BBB dysfunction is important in this context because it may occur before CNS access, during barrier crossing or bypassing, or after brain infection as part of secondary neurovascular injury. It therefore provides a practical basis for organizing unresolved questions in TMUV neuroinvasion and neuropathogenesis.

### 1.3 Blood–brain barrier dysfunction as a framework for studying Tembusu virus neuroinvasion and neuropathogenesis

For TMUV, detection of viral RNA or antigen in brain tissues, together with brain lesions and neurological signs in infected birds or experimental models, establishes that the CNS can be involved during infection (27, 28). These findings, however, do not identify the route of CNS entry or explain how brain infection progresses to neurological injury. Two related questions are particularly important: through which route TMUV reaches the CNS, and when BBB dysfunction occurs relative to early brain infection. Possible routes include the BBB and other CNS interfaces, as well as olfactory or neural pathways. Determining when barrier disruption occurs is therefore critical for distinguishing its role in viral entry from barrier damage that develops after CNS infection. Early permeability changes would be compatible with an entry-associated process, whereas disruption emerging after substantial viral replication or neuroinflammation would more likely reflect secondary injury. This distinction is especially relevant when comparing TMUV variants with different levels of CNS pathogenicity.

Viral genetic variation may underlie strain-dependent CNS-associated phenotypes in TMUV, but its effects need to be assigned to specific disease steps rather than interpreted as a single mechanism of BBB dysfunction or CNS disease severity. Structural proteins, especially the envelope protein, are plausible determinants of receptor engagement, membrane fusion, tissue tropism, and CNS access, whereas nonstructural proteins can influence replication efficiency, innate immune antagonism, endothelial activation, and inflammatory signaling (29, 30). Mutations that enhance peripheral replication or viremia may increase viral exposure to CNS interfaces, while those that improve replication in CNS-associated cells would mainly affect events after entry. Others may modify endothelial integrity, leukocyte trafficking, or local inflammation. Thus, the phenotypic effect of a TMUV mutation should be interpreted according to the stage of pathogenesis it alters, rather than being broadly attributed to increased neurovirulence or BBB dysfunction.

Against this background, this review uses BBB dysfunction as an organizing concept because the BBB is a critical neurovascular interface where peripheral infection, CNS access, vascular permeability, immune-cell trafficking, and neuroinflammatory injury converge. This approach does not assume that BBB dysfunction is the primary or exclusive route by which TMUV reaches the CNS; instead, it helps organize questions about CNS access, barrier alteration, and neurovascular injury. The BBB provides a useful framework for examining TMUV CNS infection because it links systemic infection with neurovascular changes that accompany CNS involvement. Here, BBB dysfunction is considered in relation to CNS entry and subsequent neurovascular injury, without excluding other routes of neuroinvasion. By comparing mechanisms defined in representative neurotropic flaviviruses, we summarize how endothelial activation, tight junction remodeling, matrix metalloproteinase activity, leukocyte trafficking, glial inflammatory amplification, and viral determinants may contribute to BBB dysfunction and CNS disease. These comparative insights are then used to frame three possible roles of BBB dysfunction during TMUV neuroinvasion and neuropathogenesis: an entry-associated event, a downstream consequence, or an inflammatory amplifier.

## 2 Defining CNS disease stages in BBB-centered flavivirus research

Before discussing BBB dysfunction in flavivirus infection, it is necessary to define CNS disease as a staged process rather than a single outcome. In flavivirus research, detection of virus in the brain, neurological signs, BBB permeability changes, and mortality are often reported together, but they do not necessarily reflect the same biological process. A virus may efficiently reach the CNS after peripheral infection, but cause limited pathology after entry. Conversely, a virus may be highly pathogenic after direct CNS exposure but have limited ability to access the CNS during natural or peripheral infection. Therefore, BBB-centered studies require a stage-based approach that separates CNS access, CNS cell susceptibility, disease severity after CNS access, and the broader sequence of neuropathogenesis. In TMUV studies, these terms can be easily conflated when brain viral detection, BBB permeability changes, inflammatory responses, and neurological signs are measured at similar time points. For this reason, each readout needs to be assigned to the pathogenic stage it most directly supports.

In this context, neuroinvasion, neurotropism, neurovirulence, and neuropathogenesis should be interpreted as distinct but connected disease stages. Neuroinvasion concerns whether and how a virus reaches the CNS from peripheral sites. Neurotropism concerns which CNS-associated cells and tissues can support infection once the virus encounters them. Neurovirulence concerns the severity of neurological disease after the virus has gained access to the CNS. Neuropathogenesis refers to the integrated disease process through which viral replication, BBB dysfunction, glial activation, leukocyte infiltration, neuroinflammation, and neuronal injury are connected to the development of clinical neurological disease. These stage-specific distinctions, common interpretive pitfalls in TMUV studies, and the experimental approaches needed to separate neuroinvasion, neurotropism, BBB dysfunction, inflammatory amplification, and neurovirulence are summarized in Table 1.

**Table 1:** Stage-specific framework and experimental readouts for CNS-associated flavivirus disease.

Pathogenic stage	Key biological question	Viral determinants	Host and BBB responses	Experimental readouts/assays
**Peripheral replication and viremia**	Does the virus generate sufficient systemic exposure for CNS access?	Replication efficiency; strain-dependent fitness; innate immune evasion; peripheral viral load	Peripheral innate immune activation; systemic cytokine and chemokine induction; inflammatory tone before CNS entry	Peripheral viral titers; viremia kinetics; serum cytokines/chemokines; body weight change; systemic clinical signs; survival
**CNS entry/neuroinvasion**	When and through which route does the virus reach the CNS?	Virion stability; receptor or attachment-factor engagement; barrier-cell interaction; strain-dependent CNS access potential	Early endothelial activation; initial barrier perturbation; leukocyte adhesion or transmigration; involvement of alternative CNS barriers	Timing of CNS viral detection; brain region-specific viral distribution; CSF virus; choroid plexus infection; olfactory bulb or brainstem viral signal; vascular/perivascular localization; distinguishes CNS entry routes from post-entry replication
**BBB dysfunction**	Does barrier injury precede CNS entry, follow early brain infection, or amplify CNS inflammation?	Viral protein-mediated barrier injury; direct or indirect endothelial effects; induction of inflammatory mediators	Endothelial activation; tight junction remodeling; MMP upregulation; vascular leakage; neurovascular-unit disruption	Evans blue or tracer leakage; FITC-dextran permeability; TEER; ZO-1/occludin/claudin-5 expression or localization; MMP activity; albumin leakage; compare with first CNS-positive time point to assess a possible facilitator, consequence, or amplifier role
**Brain replication and CNS infection**	Which CNS-associated cells and brain regions support viral replication?	CNS replication efficiency; CNS-associated cell susceptibility; post-entry replication capacity; innate immune antagonism; mutations associated with CNS disease severity	Viral sensing by CNS-resident cells; early glial responses; local antiviral or inflammatory signaling	Brain viral loads; infectious virus titers; RT-qPCR; immunohistochemistry; *in situ* hybridization; region-specific viral mapping; infected cell-type identification; co-localization to define infected CNS cell types and cellular tropism
**Neuroinflammation and inflammatory amplification**	Does local inflammation amplify BBB injury and tissue damage after CNS infection?	Viral induction of proinflammatory signaling; immune-modulatory viral proteins; viral persistence; strain-dependent inflammatory potential	Microgliosis; astrogliosis; leukocyte trafficking; cytokine and chemokine amplification; secondary BBB injury	Cytokine/chemokine profiles; inflammatory gene expression; microglial markers; astrocyte markers; immune-cell infiltration; flow cytometry or immunostaining of infiltrating cells; align with viral localization to assess inflammatory amplification
**Neuronal injury and neurological disease**	How do viral replication and inflammatory injury translate into neurological disease?	CNS-intrinsic neurovirulence; neuronal or CNS-cell tropism; replication-associated cytopathology; inflammatory injury potential	Neuronal degeneration; sustained neuroinflammation; glial activation; tissue injury; possible repair or irreversible damage	Neuronal injury markers; TUNEL or cell death assays; histopathological lesions; neurological scoring; motor or behavioral abnormalities; clinical signs; mortality; direct CNS inoculation helps distinguish neurovirulence from neuroinvasion

This table summarizes the major pathogenic stages linking peripheral flavivirus infection to CNS involvement and neurological disease. Each stage is organized according to the key biological question, potential viral determinants, host and BBB responses, and representative experimental readouts. The framework is intended to help distinguish neuroinvasion, BBB dysfunction, CNS replication, inflammatory amplification, and neurovirulence as related but mechanistically distinct processes. It also highlights common TMUV interpretive pitfalls, including equating brain viral detection with BBB-dependent neuroinvasion, high brain viral load with neurotropism, and neurological severity with neurovirulence, and indicates the experimental distinctions needed to separate these processes.

### 2.1 Neuroinvasion, neurotropism, neurovirulence, and neuropathogenesis as distinct disease stages

Neuroinvasion generally refers to the capacity of a virus to enter the nervous system from peripheral sites. In flavivirus research, this is usually evaluated using peripheral infection routes, followed by detection of viral RNA, infectious virus, or viral antigen in CNS or CNS-associated tissues. However, neuroinvasion does not by itself identify the route of CNS entry. Virus detected in brain tissue after peripheral infection may have entered through BBB-dependent routes, such as paracellular leakage, transcellular endothelial passage, or endothelial infection-associated dysfunction (6, 31). Alternatively, it may have reached the CNS through BBB-independent or non-classical routes, including the blood–cerebrospinal fluid barrier, choroid plexus, olfactory pathway, peripheral nerves, or infected leukocytes (31, 32).

Neurotropism and neurovirulence address different questions. Neurotropism describes the ability or preference of a virus to infect neural or CNS-associated cells, including neurons, astrocytes, microglia, brain microvascular endothelial cells, choroid plexus cells, olfactory-associated cells, or cells within specific brain regions (33). It therefore reflects cellular and tissue susceptibility rather than CNS access itself. Neurovirulence refers to the capacity of a virus to cause neurological disease after it has gained access to the CNS (34). This is often assessed using intracranial or intracerebral inoculation models, which bypass peripheral barriers and are therefore more informative for disease severity after CNS exposure than for natural neuroinvasion.

Neuropathogenesis is broader than any one of these concepts. It describes the full pathogenic sequence by which peripheral replication, viremia, CNS entry, infection of susceptible CNS-associated cells, BBB dysfunction, neuroinflammation, and neuronal injury collectively lead to clinical neurological disease (18). The relationships among these concepts and their position within the broader CNS disease sequence are illustrated in Figure 1. For TMUV, this distinction is particularly relevant because neurological signs and CNS lesions have been increasingly reported in infections caused by some recent or emerging strains, whereas it remains unclear whether BBB dysfunction facilitates CNS entry, follows early CNS infection, or amplifies neuroinflammatory injury after the virus has reached the brain.

**Figure 1:**
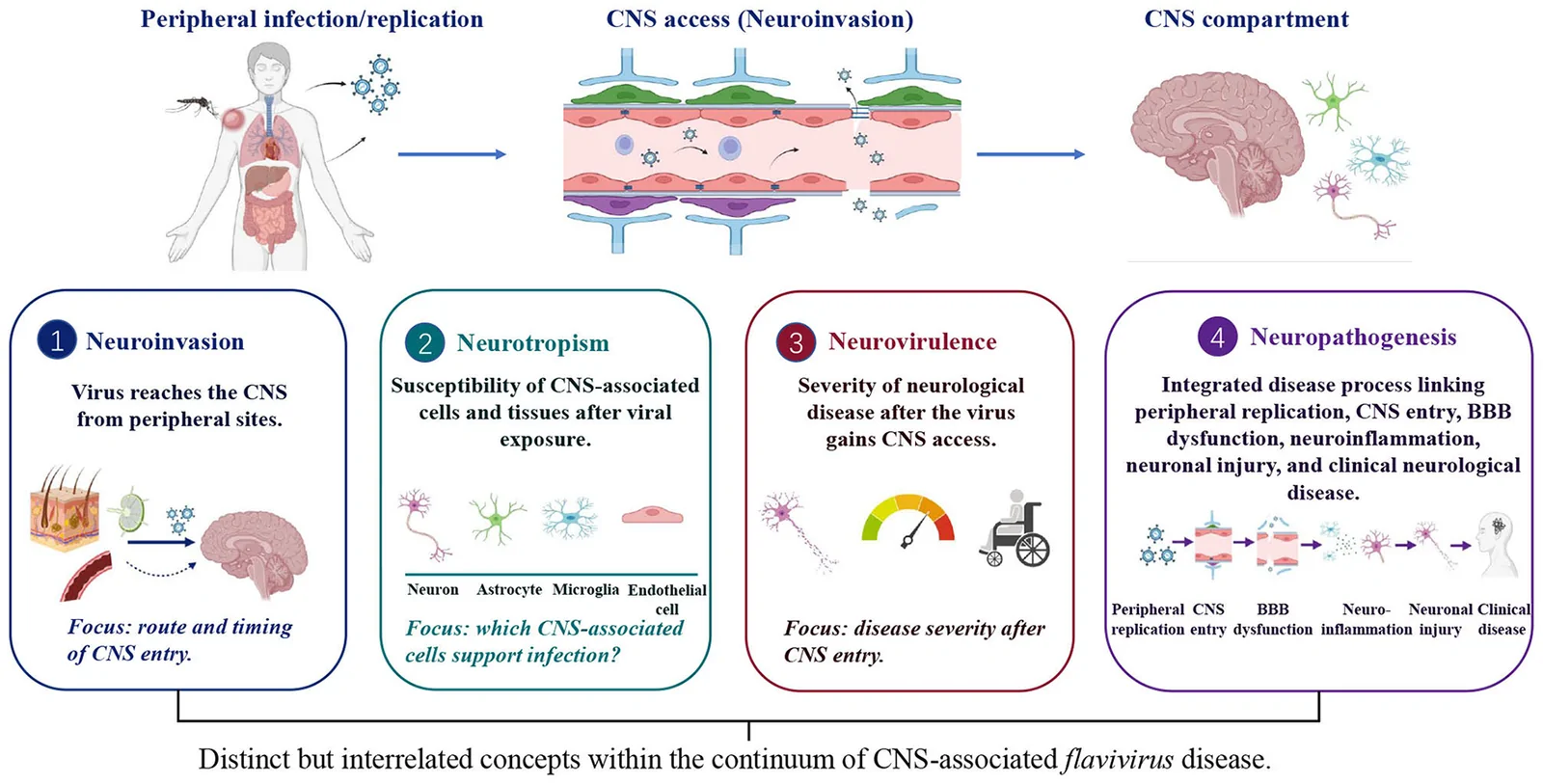
CNS-associated flavivirus disease stages and key concepts. This conceptual framework distinguishes four related but mechanistically distinct terms in flavivirus-associated CNS disease. Neuroinvasion refers to the ability of virus to reach the CNS from peripheral sites. Neurotropism refers to the susceptibility of CNS-associated cells and tissues to viral infection after the virus encounters them. Neurovirulence refers to the severity of neurological disease after the virus has gained access to the CNS. Neuropathogenesis refers to the broader integrated disease process linking peripheral replication, CNS entry, BBB dysfunction, neuroinflammation, neuronal injury, and clinical neurological disease. The figure also highlights that direct CNS inoculation is more appropriate for assessing neurovirulence than natural neuroinvasion, and that brain viral detection alone does not define the route of CNS entry.

### 2.2 Timing of BBB dysfunction in TMUV neuroinfection

For TMUV, the timing of BBB dysfunction is critical for distinguishing different mechanisms of neuroinfection. Detection of viral RNA or antigen in brain tissues, increased BBB permeability, and neurological signs may occur within the same disease course, but these observations do not necessarily indicate that BBB dysfunction is temporally upstream of CNS entry. BBB dysfunction may precede detectable brain infection and facilitate viral access to the CNS, but it may also arise after early CNS infection has been established or develop as part of a later inflammatory response. Therefore, BBB changes must be interpreted in relation to peripheral viral replication, viremia, CNS entry, local brain replication, neuroinflammation, and neurological disease progression.

This distinction is particularly relevant for recent or emerging TMUV strains in which neurological signs and CNS lesions have been increasingly reported. A stronger CNS-associated phenotype may reflect enhanced peripheral replication and higher viremia, more efficient crossing or bypassing of CNS barriers, altered tropism for CNS-associated cells, increased replication within brain tissues, stronger BBB dysfunction, or amplified inflammatory injury. Without temporal information, these possibilities cannot be distinguished. For example, BBB permeability changes detected before or at the earliest stage of brain viral positivity would support a possible role in CNS access, whereas BBB dysfunction detected after substantial brain viral replication would be more consistent with a downstream consequence of CNS infection. If BBB dysfunction coincides with leukocyte infiltration, glial activation, cytokine induction, and worsening neurological signs, it may function primarily as an amplifier of neuroinflammatory injury.

Accordingly, TMUV studies should combine time-resolved virological, BBB, inflammatory, and neurological readouts. Such studies would help determine whether BBB dysfunction is temporally upstream of CNS entry, secondary to early brain infection, or part of a feed-forward inflammatory process that exacerbates neurological disease. This timing-based analysis provides a mechanistic basis for interpreting BBB dysfunction in TMUV neuroinfection and for designing experiments that distinguish neuroinvasion, neurotropism, neurovirulence, and inflammatory neuropathogenesis.

## 3 Blood–brain barrier dysfunction in neurotropic flavivirus infection: shared mechanisms and virus-specific differences

BBB alterations are common during neurotropic flavivirus infection, although their timing, severity, and functional significance vary among viruses and experimental settings. Mechanistic studies have implicated endothelial activation, inflammatory signaling, junctional remodeling, immune-cell trafficking, and glial responses in these changes (18). These mechanisms overlap across flaviviruses, but their relative contribution differs by virus species, host, route of infection, sampling time, and experimental model. For this reason, BBB involvement is better interpreted as a group of partially shared mechanisms with virus-specific weighting, rather than as one conserved BBB-dysfunction pathway. This distinction is important for TMUV, where CNS involvement has been described but the timing and route of CNS entry remain unresolved. The major mechanistic modules of flavivirus-associated BBB dysfunction are summarized in Figure 2.

**Figure 2:**
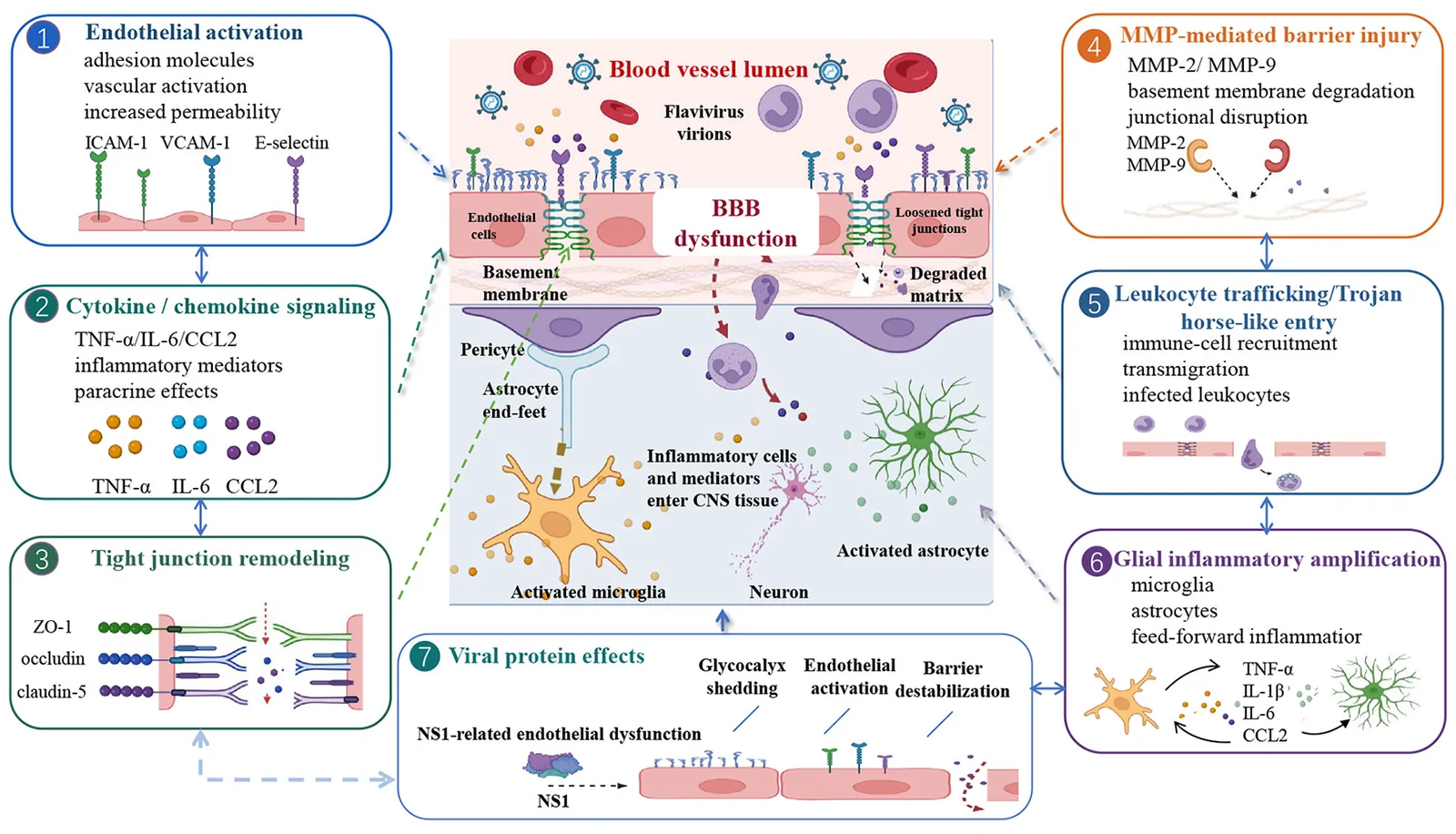
Mechanistic modules of flavivirus-associated BBB dysfunction. Flavivirus-associated BBB dysfunction does not represent a single conserved pathway, but rather a set of overlapping mechanistic modules that variably contribute across viral systems and experimental contexts. These modules include endothelial activation, cytokine and chemokine signaling, tight junction remodeling, MMP-mediated barrier injury, leukocyte trafficking or Trojan horse-like entry, glial inflammatory amplification, and viral protein-mediated endothelial dysfunction, including NS1-related effects. Together, these processes can increase vascular permeability, destabilize the neurovascular unit, and promote CNS access or neuroinflammatory injury. Representative neurotropic flaviviruses, including JEV, WNV, ZIKV, DENV, and TBEV, differentially emphasize these modules, which provide comparative reference points for TMUV rather than direct mechanistic templates.

### 3.1 BBB structure, integrity markers, and routes of CNS entry

The BBB is a specialized component of the neurovascular unit that limits uncontrolled entry of pathogens, immune cells, and circulating molecules into the CNS. Its physical barrier is formed mainly by brain microvascular endothelial cells connected by tight junctions, but its function depends on a broader cellular and extracellular environment that includes pericytes, astrocytic end-feet, basement membrane components, and immune-regulatory cells. Barrier integrity therefore cannot be inferred from endothelial survival alone. Junctional protein organization, cytoskeletal stability, vesicular and transendothelial transport, and signaling between endothelial, glial, and vascular cells all contribute to whether the BBB remains restrictive or becomes permissive during infection (35).

BBB integrity is usually assessed by combining structural markers with functional readouts. ZO-1, occludin, and claudin-5 are commonly measured by immunofluorescence, western blotting, or transcript analysis to evaluate tight junction organization (36). VE-cadherin and junctional adhesion molecules provide additional information on endothelial junctional stability and cell–cell adhesion (37). Functional assays address a different question: whether the barrier actually permits increased passage of ions, tracers, plasma proteins, or other circulating molecules. These assays include transendothelial electrical resistance, FITC-dextran or related tracer permeability, Evans blue extravasation, albumin leakage, and imaging-based assessment of vascular permeability (38). The interpretation of these readouts is stage-dependent. Redistribution or discontinuous staining of ZO-1, occludin, or claudin-5 indicates junctional remodeling, but it does not necessarily imply widespread endothelial cell death (36). Reduced transendothelial electrical resistance or increased FITC-dextran permeability provides stronger evidence of functional barrier weakening (39). *In vivo*, Evans blue extravasation or albumin leakage supports vascular leakage, but these endpoints do not distinguish direct endothelial injury from cytokine-driven activation or secondary neurovascular damage unless they are combined with time-course, localization, and inflammatory readouts (40).

Flaviviruses can reach the CNS through more than one route. BBB-associated entry may involve paracellular passage after tight junction disruption, transcellular passage across endothelial cells, or endothelial dysfunction induced by infection or inflammation (6). Other routes include the blood–cerebrospinal fluid barrier, choroid plexus, olfactory pathway, peripheral nerves, and leukocyte-associated trafficking across endothelial barriers (31, 32). Brain viral detection after peripheral infection therefore supports CNS involvement and viral access to CNS-associated tissues, but it does not by itself prove BBB-dependent neuroinvasion. The more informative question is temporal: does BBB dysfunction appear before the first detectable CNS viral signal, after early CNS infection, or during later neuroinflammatory amplification? This distinction determines whether BBB dysfunction is interpreted as a route of entry, a consequence of early brain infection, or a contributor to disease progression.

### 3.2 Endothelial activation and cytokine-mediated barrier dysfunction

Brain microvascular endothelial cells form the vascular interface of the BBB and actively regulate permeability, leukocyte adhesion, and inflammatory signaling. During flavivirus infection, their function can be altered by viral infection itself or by inflammatory mediators generated in the periphery and CNS. Direct endothelial infection has been demonstrated in some flavivirus models (41), although its contribution appears to vary among viruses and disease stages. In many settings, barrier disruption occurs with endothelial activation and junctional remodeling rather than widespread endothelial cell death (7). These changes increase vascular permeability and promote leukocyte recruitment across the BBB.

Cytokine-mediated endothelial dysfunction is a recurring mechanism in flavivirus-associated BBB injury. TNF-α, IL-1β, IL-6, and IFN-γ have all been linked to increased endothelial permeability (42, 43), while CCL2 and CXCL10 can enhance immune-cell recruitment to the neurovascular interface (44, 45). These responses may initially contribute to antiviral defense, but excessive or sustained activation can compromise BBB integrity and promote neuroinflammatory damage.

In WNV infection, peripheral replication and viremia are associated with inflammatory and endothelial responses that can alter BBB permeability and leukocyte trafficking, although the timing of these changes relative to CNS entry varies among experimental models (46). JEV provides clearer temporal evidence that BBB disruption can occur after neuroinvasion. In a mouse model, virus was detectable in the brain before measurable increases in BBB permeability, while CNS inflammation also preceded overt barrier breakdown (43). In those settings, endothelial injury is better interpreted as a downstream component of encephalitic disease rather than as the initiating route of CNS entry. ZIKV further shows that barrier interaction is only one part of CNS pathogenesis, because its neurological phenotype also depends strongly on susceptible neural-cell populations and developmental context (47). DENV is informative in another respect. It is not a classical neuroinvasive flavivirus, but severe dengue and NS1-mediated vascular leakage demonstrate how flavivirus proteins and inflammatory responses can compromise endothelial barrier function (8). DENV therefore provides mechanistic insight into endothelial permeability and viral protein-mediated barrier injury, but it should not be used as direct evidence for BBB-dependent neuroinvasion.

Taken together, current evidence indicates that endothelial involvement in flavivirus-associated BBB dysfunction differs among viral systems and may occur at distinct points during infection, rather than following a single uniform pattern. Endothelial activation may contribute to CNS access during systemic infection, arise in response to early CNS infection, or amplify neuroinflammatory injury, depending on the virus, host, infection route, and experimental model. For TMUV, it will therefore be important to determine whether BBB dysfunction reflects direct infection of brain endothelial cells, indirect cytokine-mediated activation, systemic inflammation, local CNS inflammation, or a combination of these processes.

### 3.3 Tight junction remodeling and matrix metalloproteinase-mediated barrier injury

Tight junctions between brain microvascular endothelial cells provide the main paracellular restriction of the BBB. ZO-1, occludin, and claudin-5 are therefore commonly used to evaluate BBB integrity, but changes in these proteins require careful interpretation (36). Loss of total protein abundance, redistribution away from endothelial borders, or discontinuous junctional staining may reflect different degrees of junctional remodeling rather than the same type of barrier injury. During flavivirus infection, junctional remodeling may result from inflammatory and endothelial responses, oxidative stress, or virus–cell interactions (48). Increased paracellular permeability can then enhance the passage of inflammatory mediators and leukocytes across the BBB. Whether viral particles themselves enter through this route depends on the timing of junctional injury relative to the first detectable CNS viral signal, and cannot be inferred from tight junction disruption alone.

Matrix metalloproteinases provide an additional mechanism linking inflammation to BBB structural injury. MMP-2, MMP-3, and MMP-9 can degrade extracellular matrix and basement membrane components, destabilize the neurovascular unit, and contribute to junctional dysfunction (49). In viral encephalitis models, increased MMP activity has been associated with vascular leakage, immune-cell infiltration, and aggravated CNS inflammation (50). Importantly, MMP activation in neurotropic flavivirus infection should not be interpreted solely as a direct consequence of viral infection of endothelial cells. Infiltrating leukocytes, activated endothelial cells, astrocytes, and microglia may also produce MMPs during neuroinflammation. In this setting, MMPs provide a mechanistic link between inflammatory-cell recruitment, glial activation, and secondary BBB structural injury (51).

Evidence for tight junction remodeling and MMP-mediated barrier injury is strongest in WNV and JEV models, whereas DENV and ZIKV provide more context-specific evidence for endothelial barrier dysfunction. In WNV infection, both *in vitro* and *in vivo* studies have shown dysfunction of BBB-associated junctional complexes. WNV infection of human brain microvascular endothelial cells modulates tight junction proteins and cell adhesion molecules and facilitates leukocyte transmigration across an *in vitro* BBB model. In mice, WNV-induced BBB dysfunction is characterized by reduced levels of junctional complex proteins, including ZO-1, occludin, claudin-1, JAM-A, β-catenin, and VE-cadherin, together with increased MMP-1, MMP-3, and MMP-9 and leukocyte infiltration in the brain. Importantly, MMP inhibition can reverse WNV-induced BBB dysfunction and tight junction protein degradation, supporting a mechanistic link between MMP activation, junctional injury, and BBB leakage in WNV infection (7, 46, 52).

JEV also provides strong evidence that tight junction alteration contributes to BBB dysfunction, but the mechanism often appears to involve inflammatory activation of the neurovascular unit rather than simple direct endothelial cytolysis. JEV infection has been associated with reduced expression or altered integrity of tight junction proteins such as claudin-5, ZO-1, and occludin, increased BBB permeability, and neuroinflammatory injury. In addition, JEV-infected pericytes can release IL-6 and induce ZO-1 degradation in brain microvascular endothelial cells, while JEV-infected astrocytes can disrupt endothelial barrier integrity through release of MMP-2, MMP-9, IL-6, and VEGF (53, 54). These findings indicate that JEV-associated BBB dysfunction can arise through multicellular neurovascular signaling, in which infected or activated pericytes and astrocytes contribute to endothelial tight junction remodeling and MMP-mediated barrier injury.

Although DENV is not classically used as a model of BBB-dependent neuroinvasion, it provides important evidence that flavivirus proteins can compromise endothelial barrier stability through host protease-dependent mechanisms. In particular, DENV NS1 can induce MMP-9 expression and facilitate MMP-9-mediated dysfunction of adhesion and tight junction proteins, including β-catenin, ZO-1, and ZO-2, thereby promoting endothelial hyperpermeability and vascular leakage (55). Therefore, DENV is highly informative for understanding how viral proteins and host proteases affect endothelial barriers, but these findings mainly reflect systemic vascular leakage and should be applied cautiously to CNS-specific BBB dysfunction.

ZIKV shows that CNS barrier interaction does not necessarily require overt BBB breakdown. In several experimental models, the virus can infect or traverse barrier-associated cells and alter junctional or barrier properties (47). Other BBB models, however, have demonstrated viral crossing with little measurable loss of barrier integrity (56). Barrier passage and barrier disruption should therefore be considered separately in ZIKV infection. Its neurological disease also depends strongly on neural progenitor susceptibility, developmental stage, and placental–fetal infection, making BBB injury only one component of a broader pathogenic process.

The contribution of tight junction remodeling and MMP activity therefore differs substantially among flaviviruses. In WNV, these changes often accompany BBB leakage and leukocyte infiltration, whereas in JEV they may emerge as part of the inflammatory injury that follows CNS infection. DENV provides strong evidence for protease- and junction-related vascular dysfunction, but mainly in the setting of systemic vascular leakage rather than neuroinvasion. ZIKV further separates barrier crossing from overt barrier disruption. For TMUV, junctional proteins and MMPs are therefore most informative when interpreted together with infection kinetics. Changes detected at or before the earliest CNS-positive stage would be consistent with an entry-associated role, whereas later changes would favor secondary neurovascular injury.

### 3.4 Leukocyte trafficking, Trojan horse entry, and glial inflammatory amplification

Leukocyte recruitment is a prominent feature of flaviviral neuroinflammation. Endothelial activation and chemokine signaling promote immune-cell adhesion and migration across CNS barriers, where infiltrating cells may contribute to viral control or exacerbate local inflammation and barrier injury (57). In some infections, leukocytes can also carry infectious virus into the CNS, providing a potential Trojan horse-like route of dissemination (58). However, leukocyte accumulation in infected brain tissue alone does not establish this mechanism, particularly when infiltration occurs after CNS infection is already detectable.

WNV provides some of the strongest evidence that leukocyte trafficking can have distinct roles during CNS infection. *In vivo*, CCR5-dependent leukocyte recruitment contributes to viral control and survival (59), whereas WNV-infected neutrophils can enter the CNS through an osteopontin-dependent pathway and increase brain viral burden and disease severity (60). These findings distinguish protective immune recruitment from leukocyte-associated viral dissemination and show that their effects depend on the recruited cell population and stage of infection.

In JEV infection, leukocyte infiltration is more consistently associated with encephalitic inflammation than with proven leukocyte-mediated neuroinvasion. Leukocyte accumulation accompanies glial activation, cytokine production, and neuronal damage (61), and in experimental models much of this response develops after CNS infection has begun (43). Activated microglia and astrocytes can then reinforce local inflammation and endothelial dysfunction through cytokine and chemokine signaling. Thus, JEV provides stronger evidence for leukocyte–glia–endothelium amplification of CNS injury than for a defined Trojan horse route of entry.

DENV is less informative for CNS entry but illustrates how infected or activated leukocytes can contribute to vascular dysfunction. Monocytes and macrophages are major participants in dengue immunopathology, and their interaction with activated endothelium can promote inflammatory signaling and vascular leakage (62). Although infected leukocytes have been discussed as vehicles of viral dissemination, these observations do not provide direct evidence for leukocyte-mediated BBB crossing in dengue.

Evidence for a dominant leukocyte-driven mechanism is also limited in ZIKV infection. Immune-cell responses can contribute to inflammatory pathology, but neurological disease is strongly influenced by developmental stage, placental–fetal infection, and the susceptibility of neural progenitor and other CNS-associated cells (16). Leukocyte trafficking is therefore better viewed as one component of ZIKV-associated inflammation rather than a defining route of neuroinvasion.

Once infection or inflammatory signaling is established within the CNS, resident glial cells can further amplify neurovascular injury. Microglia and astrocytes respond to viral and inflammatory stimuli by releasing cytokines, chemokines, reactive mediators, and MMPs that can alter endothelial function, recruit additional leukocytes, and contribute to neuronal damage (63). This creates a reinforcing interaction among glial activation, leukocyte recruitment, endothelial dysfunction, and BBB injury. For TMUV, the timing of leukocyte infiltration and glial activation will therefore be critical: early association with CNS entry may support a role in viral dissemination, whereas later accumulation would be more consistent with neuroinflammatory amplification and secondary barrier damage.

### 3.5 Lessons from flavivirus BBB dysfunction for studying TMUV-associated CNS disease

Comparative studies of flaviviruses show that BBB dysfunction can arise at different stages of CNS disease and through different mechanisms. WNV links systemic infection with neurovascular and leukocyte responses associated with CNS invasion, whereas JEV more clearly demonstrates barrier injury during established encephalitis. DENV defines mechanisms of endothelial leakage that are relevant to vascular injury but not necessarily to neuroinvasion, while ZIKV shows that CNS barrier interaction can occur without overt barrier breakdown and must be considered alongside neural-cell and developmental susceptibility.

For TMUV, the main value of these comparisons is to establish the timing and context of BBB-related changes. Endothelial or junctional alterations detected around the onset of CNS infection would warrant investigation for a possible role in viral access, whereas changes emerging after substantial brain infection would more likely reflect neurovascular injury or inflammatory amplification. Evidence of virus-bearing leukocytes crossing CNS barriers would be needed to support cell-associated entry, while glial and cytokine responses are more informative for events occurring after CNS infection is established. These comparative patterns and their implications for TMUV research are summarized in Table 2. They also provide the rationale for examining viral determinants that may influence cell entry, tissue tropism, replication efficiency, endothelial interaction, and immune modulation in the following section.

**Table 2:** Comparative mechanisms of flavivirus-associated BBB dysfunction and implications for TMUV research.

Virus	BBB-related emphasis in this review	Major mechanistic modules	Stage most strongly informed	Implications for TMUV research
**Japanese encephalitis virus (JEV)**	Encephalitis-associated BBB dysfunction closely linked to neuroinflammation and secondary neurovascular injury	Cytokine-mediated endothelial activation; tight junction alteration; MMP activity; glial activation; leukocyte infiltration; pericyte- or astrocyte-mediated endothelial injury	Downstream BBB injury after CNS infection; inflammatory amplification; neurovascular damage associated with CNS disease severity	Test whether TMUV BBB dysfunction follows early CNS infection and whether glial activation, cytokines, chemokines, and MMP activity drive secondary barrier injury
**West Nile virus (WNV)**	Well-characterized example linking peripheral infection, viremia, endothelial activation, leukocyte trafficking, BBB permeability, and CNS invasion	Endothelial activation; chemokine induction; adhesion molecule upregulation; leukocyte transmigration; tight junction disruption; MMP-mediated barrier injury; possible Trojan horse-like entry	BBB-associated neuroinvasion; leukocyte-associated CNS entry; transition from peripheral infection to CNS involvement	Evaluate whether TMUV strains with high or sustained viremia use endothelial activation, leukocyte trafficking, or BBB-associated routes to enhance CNS access
**Zika virus (ZIKV)**	Barrier interaction and CNS disease shaped by developmental context and neural-cell susceptibility rather than a uniform BBB-disruption mechanism	Interaction with CNS barrier cells; trans-barrier passage in some models; neural progenitor susceptibility; placental-fetal and developmental interfaces	Barrier interaction; CNS-associated cell susceptibility; developmental neuropathogenesis	Avoid assuming that TMUV brain infection requires overt BBB disruption; evaluate CNS-associated cell susceptibility, host species, and developmental or age-related context
**Dengue virus (DENV)**	Endothelial dysfunction and vascular leakage, especially through NS1-related mechanisms; not a classical BBB-dependent neuroinvasion model	NS1-mediated endothelial glycocalyx disruption; MMP-9 induction; tight/adherens junction alteration; inflammatory vascular injury; endothelial hyperpermeability	Endothelial barrier biology; viral protein-mediated vascular dysfunction; systemic permeability rather than direct BBB-dependent neuroinvasion	Use DENV mainly as a reference for viral protein-mediated endothelial dysfunction and barrier permeability, not as direct evidence for TMUV BBB-dependent CNS entry
**Tick-borne encephalitis virus (TBEV)**	Classical neurotropic flavivirus comparator, but BBB-related mechanisms are less specifically developed in the current TMUV framework	Possible CNS barrier involvement; inflammatory neurovascular injury; unresolved route and timing of BBB contribution	Neurotropic CNS disease with remaining uncertainty about BBB involvement	Use TBEV cautiously as a neurotropic comparator; do not infer a specific TMUV BBB-entry mechanism without time-resolved and route-specific evidence
**Tembusu virus (TMUV)**	Emerging avian flavivirus with increasing CNS-associated phenotypes; BBB timing, entry routes, and strain-specific mechanisms remain unresolved	Candidate mechanisms include endothelial activation, tight junction/MMP-mediated injury, leukocyte-associated CNS entry, glial inflammatory amplification, alternative CNS entry routes, and viral determinant-driven stage-specific effects	Open question: BBB-associated CNS entry, downstream BBB injury, inflammatory amplification, or combined functional roles	Apply a stage-specific framework to determine whether BBB dysfunction facilitates CNS entry, follows early CNS infection, amplifies neuroinflammatory injury, or combines these roles in a strain- and host-dependent manner

This table summarizes how representative flaviviruses inform different BBB-related mechanisms and pathogenic stages. These virus-specific patterns should be used as comparative reference points for TMUV research rather than treated as direct mechanistic templates.

## 4 Viral determinants influencing flavivirus tissue distribution, CNS access, barrier interaction, and neuropathogenesis

BBB responses during flavivirus infection are shaped not only by the host environment but also by viral genetic variation. Viral determinants can influence the amount of virus reaching CNS interfaces, its ability to enter or replicate in CNS-associated cells, and the inflammatory response that follows infection. Their effects therefore depend on where they act along the course of infection. The following sections focus on the envelope protein and selected nonstructural proteins, which provide the clearest examples of viral factors affecting tissue distribution, CNS access, barrier interaction, and subsequent neuropathogenesis.

### 4.1 Envelope protein determinants of viral entry, tissue tropism, and CNS access

The flavivirus envelope (E) protein governs several early steps of infection, including receptor attachment, endocytic uptake, membrane fusion, and virion stability, and is also the principal target of neutralizing antibodies (64). Changes in E protein sequence or post-translational features, such as glycosylation, can alter receptor engagement, fusion efficiency, pH-dependent conformational changes, and virion stability, thereby influencing which host cells and tissues support infection (65). In neurotropic flaviviruses, these changes may affect peripheral replication, systemic dissemination, interaction with CNS barrier-associated cells, CNS access, infection of neural or CNS-associated cells, and ultimately disease severity.

WNV provides representative evidence that E protein determinants can influence both CNS access and neurological disease severity. A mutation in the E protein fusion loop of the NY99 strain was shown to attenuate mouse neuroinvasiveness, directly linking an entry-related region of the E protein to CNS disease after peripheral infection (66). Separately, amino acid 159 of the E protein has been implicated in strain-dependent differences in neurovirulence in mice, with effects on viral replication and inflammatory-cell infiltration after intracranial inoculation (67). These findings indicate that WNV E protein variation can influence distinct stages of CNS pathogenesis, including neuroinvasion after peripheral infection and disease severity after CNS exposure.

JEV illustrates how E protein mutations can affect both CNS access and disease severity after CNS infection. In the attenuated SA14-14-2 vaccine strain, E protein mutations including L107F and E138K have been implicated in reduced neurovirulence compared with the virulent parental SA14 strain (68). This example is most relevant to neurovirulence, because the mutations affect disease severity after the virus reaches or is introduced into the CNS. In contrast, recent work on the JEV E389 mutation suggests that altered E protein flexibility and increased sensitivity to heparin inhibition may reduce viral entry into brain tissues, thereby attenuating neuroinvasiveness (69). This finding is more closely related to attachment-factor engagement and CNS access. Thus, the SA14-14-2 mutations and E389 affect different stages of CNS disease: the former are linked mainly to neurovirulence, whereas E389 is more closely associated with neuroinvasion.

In ZIKV, E protein variation can affect systemic spread, fetal tissue access, and CNS-associated disease through different mechanisms. The E-V473M mutation has been reported to enhance viremia, maternal-to-fetal transmission, and CNS-associated disease severity (70), suggesting an effect on systemic dissemination and access to developmentally vulnerable tissues. E-D67N, by contrast, has been associated with more severe neurolesions after infection is established (71). E protein glycosylation contributes to attachment and infection of lectin-expressing cells, adding a cell-entry and tissue-distribution component to ZIKV pathogenesis (72). These observations separate determinants that act mainly through dissemination and tissue access from those associated with infection of susceptible neural or developmental compartments and subsequent CNS injury.

Together, evidence from WNV, JEV, and ZIKV supports the view that E protein determinants influence CNS disease through multiple but distinguishable stages. Some E protein changes may primarily affect viral particle stability, receptor or attachment-factor engagement, and systemic dissemination before CNS access. Others may alter interaction with CNS barrier-associated cells, infection of neural or CNS-associated cells, replication after CNS entry, or disease severity. Therefore, E protein determinants should be interpreted according to their position in the peripheral-to-CNS disease sequence, rather than described broadly as determinants of neuroinvasion, neurotropism, or neurovirulence.

### 4.2 Nonstructural proteins in barrier interaction, viral replication, and inflammatory neuropathogenesis

Unlike the envelope protein, which directly controls viral attachment, fusion, and entry, flavivirus nonstructural proteins usually influence CNS disease through post-entry and host-response mechanisms (73). Through effects on viral replication, innate immune signaling, endothelial responses, and inflammation, these proteins can modify both systemic infection and events that follow CNS entry (74–76).

NS1 is the clearest example of a nonstructural protein with direct relevance to barrier biology because it is secreted from infected cells and can act on endothelial surfaces independently of direct virion entry. In DENV infection, soluble NS1 has been shown to disrupt the endothelial glycocalyx by inducing degradation of sialic acid and shedding of heparan sulfate proteoglycans, leading to endothelial hyperpermeability (77). DENV NS1 can also trigger vascular leakage through endothelial cell-intrinsic mechanisms that are at least partly independent of TNF-α and IL-6, indicating that NS1-mediated barrier injury is not simply a secondary consequence of inflammatory cytokine production (78). Together, these findings establish NS1 as a soluble viral factor capable of directly altering vascular barrier function.

NS1-related endothelial dysfunction should not be treated as identical across flaviviruses or vascular beds. Comparative studies have shown that NS1 proteins from DENV, ZIKV, WNV, JEV, YFV, and related flaviviruses can induce tissue-specific endothelial dysfunction, with patterns that partly reflect viral disease tropism (8). These effects differ among viruses and tissues. In WNV infection, circulating NS1 levels have been associated with viral dissemination to the brain (79). JEV provides more direct evidence at the BBB: secreted NS1 and the JEV-serogroup-specific NS1′ increase the permeability of brain microvascular endothelial cells and promote tight junction protein degradation through an autophagy–lysosomal pathway, with NS1′ exerting the stronger effect (80). Together, these studies indicate that NS1-mediated barrier effects are tissue dependent rather than uniform across flaviviruses.

Other nonstructural proteins are more likely to influence CNS disease by altering replication fitness and antiviral signaling than by acting directly on endothelial barriers. NS3 and NS5 are central components of the flavivirus replication machinery, and NS5 also functions as a major antagonist of type I interferon signaling. In JEV infection, NS5 inhibits IFN-stimulated JAK–STAT signaling, including STAT1 activation and nuclear translocation (81). Additional work shows that JEV NS5 can suppress dsRNA-induced IFN-β expression, indicating interference with antiviral signaling upstream of interferon production (82). Such effects may influence CNS disease indirectly by sustaining viral replication or delaying innate immune control. Prolonged viremia can increase the duration of viral exposure at CNS interfaces, whereas enhanced replication after entry can increase viral burden within CNS-associated cells. NS3 and NS5 are therefore more relevant to replication fitness and antiviral immune evasion than to direct BBB injury.

Taken together, nonstructural proteins thus influence flavivirus CNS disease through several mechanistically distinct routes. NS1-family proteins can act extracellularly on vascular or neurovascular barriers, whereas NS3 and NS5 more commonly alter replication fitness and antiviral immunity. These effects may occur before CNS entry by increasing viral burden or prolonging viremia, or later by supporting local replication and inflammatory responses within the CNS. For TMUV, however, NS1-, NS3-, and NS5-related mechanisms remain cross-flavivirus leads rather than established explanations for CNS entry or BBB dysfunction.

### 4.3 Linking candidate viral determinants to stages of Tembusu virus CNS disease

For TMUV, the main lesson from other flaviviruses is that viral genetic determinants should be assigned to defined stages of CNS pathogenesis rather than treated as general virulence markers. Structural protein variation, nonstructural protein changes, or individual amino acid substitutions could intensify CNS-associated disease through different routes. One determinant may increase peripheral replication and viremia before the virus reaches CNS barriers. Another may alter interaction with endothelial cells, choroid plexus-associated cells, olfactory-associated tissues, or other entry-related interfaces. Other changes may act predominantly after CNS entry by enhancing replication in susceptible cells or local inflammatory responses. The key issue is therefore which stage of the peripheral-to-CNS sequence is altered, from peripheral replication and viremia to CNS access, post-entry replication, BBB dysfunction, and neuroinflammatory injury.

Structural determinants in the C-prM-E region, particularly the envelope protein, are most likely to affect viral entry and tissue distribution through changes in receptor engagement, membrane fusion, virion stability, and cell tropism (29, 83). In TMUV, these changes could contribute to CNS-associated pathogenicity in several non-equivalent ways. If they improve replication in peripheral tissues, they may increase viremia and raise the amount of virus reaching CNS barriers. If they alter interaction with endothelial cells, olfactory-associated cells, choroid plexus-associated cells, or other barrier-related targets, they may affect CNS access. If they expand the range of susceptible CNS-associated cells or improve replication after entry, they would be more relevant to CNS-associated cell tropism, post-entry brain replication, or disease severity after CNS access. These effects should therefore be distinguished according to whether they act before CNS entry, at CNS interfaces, or after infection is established within the brain.

Nonstructural determinants are likely to contribute through different, often indirect, mechanisms. Changes in NS1, NS3, NS4, NS5, or untranslated regions may alter replication fitness, type I interferon antagonism, host adaptation, cytokine induction, endothelial activation, or inflammatory signaling (30, 84, 85). These effects can increase CNS involvement without necessarily acting through direct BBB disruption. Enhanced replication or stronger innate immune antagonism may increase peripheral viral burden, prolong viremia, and extend viral exposure at CNS interfaces. After CNS infection is established, inflammatory signaling may instead contribute to endothelial dysfunction, leukocyte recruitment, MMP activity, glial activation, and secondary BBB injury. In this sense, nonstructural determinants may influence TMUV neuroinvasion and neuropathogenesis upstream of CNS entry, at the barrier interface, or after brain infection has been established.

These distinctions provide a practical basis for TMUV-focused pathogenesis studies. Candidate structural or nonstructural determinants should not be evaluated only by mortality, total brain viral load, or gross disease severity. These endpoints are useful, but they do not reveal which step of the disease sequence has changed. Higher brain viral loads, for example, may result from greater peripheral replication, more efficient CNS access, enhanced post-entry replication, impaired antiviral control, or secondary barrier injury. The following section therefore applies this evidence hierarchy to TMUV-specific questions about CNS involvement, BBB dysfunction, viral determinants, and host inflammatory responses. Consistent with the evidence hierarchy summarized in Table 3, current TMUV data more directly implicate E-protein residues in CNS-associated phenotypes. By contrast, NS protein-mediated IFN antagonism remains a plausible indirect contributor rather than an established mechanism of TMUV CNS entry or BBB dysfunction.

**Table 3:** Evidence hierarchy and unresolved questions for TMUV-associated CNS involvement and BBB dysfunction.

TMUV-related question or process	Current evidence in TMUV	Evidence from other flaviviruses	Current interpretation for TMUV	Key validation steps needed
**CNS involvement during TMUV infection**	Neurological signs, brain lesions, viral RNA or antigen detection in brain tissues, and nonsuppurative encephalitis have been reported in several TMUV infection settings	JEV, WNV, ZIKV, and TBEV provide established examples of flavivirus-associated CNS disease, including encephalitis, neuronal injury, and neurological dysfunction	Current TMUV evidence supports CNS involvement, but brain viral detection alone does not define the route of CNS entry, the infected CNS-associated cell types, or the mechanism of neurological injury	Perform time-course studies after peripheral infection; map the earliest CNS-positive sites; combine viral RNA, infectious virus, antigen localization, histopathology, and neurological scoring
**Timing of BBB dysfunction relative to CNS infection**	A duckling infection study showed that TMUV could be detected in brain tissue before overt BBB disruption became apparent, indicating that overt BBB disruption was more likely a downstream event after early CNS infection in that model	In some JEV models, CNS infection and neuroinflammation can precede overt BBB disruption. In WNV infection, BBB permeability and endothelial activation are closely linked to the transition from peripheral infection to CNS involvement	Available TMUV evidence supports a ‘CNS infection first, overt BBB disruption later' temporal pattern in at least one duckling model, but this finding should not be generalized to all TMUV infection contexts. BBB dysfunction may show different timing in other TMUV lineages, host ages, host species, or routes of infection	Extend time-course analyses to representative TMUV lineages, host ages, host species, and infection routes; compare viremia, first CNS viral detection, BBB permeability, tight junction markers, endothelial activation, and inflammatory readouts to determine whether post-entry BBB dysfunction is a general pattern or a model-specific finding
**Functional roles of BBB dysfunction in TMUV CNS disease**	TMUV studies have reported CNS infection and, in some cases, BBB dysfunction or permeability changes, but current evidence does not establish whether BBB dysfunction facilitates CNS entry, follows early CNS infection, or amplifies neuroinflammatory injury	WNV supports a possible role in which the BBB/neurovascular interface contributes to viral CNS entry; JEV supports inflammatory BBB injury after CNS infection; DENV NS1 studies support viral protein-mediated endothelial dysfunction; ZIKV shows that viruses can interact with barrier structures without necessarily causing overt BBB disruption	For TMUV, the facilitator/consequence/amplifier framework should be interpreted as three possible functional roles rather than established mechanisms. Current evidence is insufficient to define a dominant BBB impairment mode across TMUV lineages	Compare BBB readouts before, at, and after the first CNS-positive time point; determine whether BBB changes correlate more closely with viremia, brain viral load, local inflammatory responses, or neurological severity under defined strain, host age, host species, and infection-route conditions
**Potential CNS entry interfaces beyond overt BBB disruption**	In a duckling model, TMUV was detected in brain before overt BBB disruption, indicating that the first CNS-positive signal did not require gross BBB breakdown. TMUV infection has also been associated with BMEC injury and perivascular/neurovascular changes, supporting possible neurovascular-interface involvement. Other CNS interfaces remain insufficiently compared	Flaviviruses can use multiple CNS entry interfaces depending on virus and model, including BBB-associated endothelial interaction, blood-CSF barrier or choroid plexus routes, leukocyte-associated transport, and, in some neurotropic viral infections, olfactory or neural pathways	Current evidence indicates that TMUV can access the CNS before overt BBB disruption, with possible neurovascular/BMEC involvement. Thus, TMUV neuroinvasion may be associated with the BBB/neurovascular interface but not necessarily dependent on disruptive BBB opening; non-BBB routes require direct comparison in the same infection models	Compare neurovascular localization with non-BBB interfaces in the same time-course models: BMEC infection, vascular/perivascular viral signal, CSF virus, choroid plexus infection, olfactory epithelium or olfactory bulb involvement, brainstem or peripheral nerve signals, and leukocyte-associated viral RNA or antigen
**Viral genetic determinants of CNS-associated phenotypes**	Some TMUV lineages or strains show stronger CNS-associated phenotypes, but the causal mutations and affected disease stages remain incompletely defined	E protein mutations in WNV, JEV, and ZIKV can affect viral entry, dissemination, tissue access, or neurovirulence. NS proteins in other flaviviruses, especially NS5 and NS1-family proteins, can influence replication fitness, IFN signaling, endothelial responses, and inflammatory disease	Current TMUV evidence mainly links CNS-associated phenotypes to specific E-protein residues with stage-dependent effects, including viremia, CNS access, post-entry CNS disease severity, mammalian adaptation, and brain inflammation. NS protein-mediated IFN antagonism is better viewed as a possible indirect contributor, rather than a primary explanation for TMUV CNS entry, BBB dysfunction, or neurovirulence	Use infectious clones, site-directed mutagenesis, rescued viruses, and matched comparative infection models; assess viremia, CNS access, brain replication, BBB status, endothelial activation, and neuroinflammation, and evaluate IFN/ISG responses when NS protein or immune-antagonism hypotheses are being tested, to assign candidate mutations to defined pathogenic stages
**Neuroinflammation and secondary BBB injury**	TMUV infection can be associated with inflammatory responses and CNS lesions, but the temporal sequence linking viral replication, glial activation, leukocyte infiltration, BBB injury, and neuronal damage remains incompletely defined	JEV and WNV show strong links among glial activation, cytokine and chemokine induction, leukocyte infiltration, MMP activity, BBB injury, and neuronal damage	In TMUV infection, inflammation may amplify BBB damage and neurological injury after CNS infection has begun, but it should not be interpreted as direct evidence of the initial CNS entry route	Combine viral localization with cytokine/chemokine profiling, microglial and astrocytic markers, immune-cell infiltration, MMP activity, BBB leakage, neuronal injury markers, and neurological scoring across time

This table distinguishes findings directly supported by TMUV studies from mechanisms mainly inferred from other flavivirus systems. It also summarizes cautious interpretations for TMUV and the key validation steps required to determine whether BBB dysfunction represents an entry-associated event, a downstream consequence of CNS infection, an inflammatory amplifier, or a phenotype associated with specific viral lineages, hosts, or infection conditions.

## 5 Implications for Tembusu virus neuroinvasion and neuropathogenesis

The mechanisms described above show that BBB involvement in flavivirus CNS disease varies with the virus, host, and stage of infection. For TMUV, the key issue is therefore to determine which of these processes are supported by direct evidence and where they occur in the course of infection.

### 5.1 Evidence for CNS involvement in Tembusu virus infection

TMUV was initially recognized mainly as a waterfowl pathogen associated with egg-drop disease, growth retardation, systemic infection, and economic losses in duck and goose production. However, accumulating evidence indicates that CNS involvement may represent an important component of TMUV pathogenicity in certain infection settings. Current evidence for TMUV CNS involvement mainly comes from neurological signs, detection of viral RNA or antigen in brain tissues, histopathological brain lesions, nonsuppurative encephalitis, inflammatory responses, and experimental infection studies in birds and mammalian models (27, 28, 86). Together, these observations extend TMUV pathogenicity beyond its classical reproductive and systemic manifestations.

The presence of TMUV RNA, infectious virus, or viral antigen in brain tissues establishes CNS involvement, but it does not by itself define the route of CNS entry, the infected CNS-associated cell types, or the mechanism of neurological injury. Neurological signs and brain lesions likewise document CNS disease without identifying the route of neuroinvasion or the relative contributions of viral replication and host injury. Some emerging or variant TMUV strains have been associated with more severe disease or stronger CNS-related phenotypes than earlier classical strains (11, 87), but the biological basis of these differences remains unclear. Clinical severity and brain viral burden alone cannot determine whether the underlying difference lies in CNS access, replication after entry, or the subsequent host response. Comparisons between classical and emerging strains should combine defined infection routes with time-resolved virological, BBB, inflammatory, and neuropathological readouts.

Thus, TMUV CNS involvement is well supported, whereas the sequence linking peripheral infection to CNS entry and neurological injury remains poorly defined. Unlike JEV and WNV, for which the major steps of neuroinvasion and neuroinflammatory disease have been studied in greater mechanistic detail, TMUV still lacks a clear temporal map connecting viremia, CNS entry, BBB status, brain replication, and inflammatory injury. The central unresolved question is how TMUV reaches the CNS and what happens after entry.

These unresolved issues provide the basis for an evidence hierarchy for TMUV-associated CNS disease. Table 3 summarizes the TMUV-specific evidence, cross-flavivirus inferences, and remaining questions that guide the following discussion of BBB dysfunction, CNS entry interfaces, viral genetic determinants, and neuroinflammatory amplification.

### 5.2 BBB dysfunction in TMUV infection: facilitator, consequence, or amplifier?

As outlined in Table 3, the unresolved issue for TMUV-associated CNS disease is not only whether BBB dysfunction occurs, but where it falls within the infection sequence. Accordingly, the facilitator, consequence, and amplifier roles discussed below should be viewed as possible roles of BBB dysfunction rather than established TMUV mechanisms. Current TMUV studies have reported brain viral detection, neurological signs, histopathological lesions, inflammatory responses, and, in some cases, BBB disruption or permeability changes. These observations establish CNS involvement, but they do not distinguish BBB-dependent entry from barrier injury that develops after early brain infection or from vascular leakage that accompanies later neuroinflammation. The timing of BBB changes relative to viremia and the first detectable CNS infection is therefore central to their interpretation.

BBB dysfunction would support an entry-associated role if permeability or junctional changes emerge during systemic infection and coincide with, or precede, the earliest CNS-positive signal. High or sustained viremia could increase viral exposure at the neurovascular interface and promote endothelial activation before brain infection becomes established (6). By contrast, BBB changes detected only after substantial CNS viral accumulation would be less consistent with a primary role in entry.

TMUV-specific evidence also supports the possibility that BBB injury follows CNS entry. This interpretation is supported by a duckling infection study in which TMUV was detected in the brain before overt BBB disruption became apparent (27). This temporal pattern suggests that early CNS access can occur without marked barrier breakdown. Alternative routes, including the blood–cerebrospinal fluid barrier, olfactory or neural pathways, leukocyte-associated transport, or limited endothelial crossing, remain possible. Once infection is established in the CNS, local viral replication and inflammatory responses may then impair BBB integrity. Under this interpretation, BBB dysfunction is a consequence of early CNS infection rather than the initiating route of CNS entry.

BBB dysfunction may also amplify neuroinflammatory injury after CNS infection is established. Increased vascular permeability can facilitate further leukocyte entry and exposure of CNS tissues to circulating inflammatory mediators. The resulting endothelial and glial activation can intensify local inflammation and neuronal injury, making barrier dysfunction a contributor to disease progression rather than necessarily to initial neuroinvasion.

These three roles should not be treated as mutually exclusive. During a single TMUV infection course, BBB dysfunction could be absent at the first CNS-positive time point, become detectable during local inflammatory activation, and later intensify immune-cell entry or tissue injury. Strain, host species, age, infection route, and sampling window may shift which role is most apparent. The most informative comparison is therefore between the timing of BBB dysfunction and the major virological and inflammatory milestones of infection. At present, however, available evidence is insufficient to define a dominant BBB impairment mode across TMUV lineages, host species, ages, or infection routes.

### 5.3 Viral genetic variation as a candidate driver of TMUV CNS-associated phenotypes

TMUV genetic divergence is a likely source of strain-dependent differences in CNS-associated pathogenicity, especially for emerging or non-classical lineages in which neurological signs, brain lesions, or higher CNS viral loads have been reported. The relevant variation may occur in structural proteins, nonstructural proteins, or other genomic regions (88). Envelope variation deserves particular attention because several entry- and tropism-related functions are concentrated in the E protein. However, associations between TMUV sequence variation and CNS phenotypes remain largely descriptive until individual changes are tested by reverse genetics in comparable infection models.

Structural protein variation provides one route by which TMUV genetic divergence could translate into CNS-associated phenotypes. Changes in the C-prM-E region may affect viral entry, particle stability, receptor or attachment-factor engagement, and tissue distribution, thereby altering both systemic exposure to CNS barriers and the ability of CNS-associated cells to support infection (29, 89). Associations between structural protein variation and higher brain viral burden or neurological disease do not identify the affected step of infection. Such phenotypes may arise from altered systemic dissemination, CNS access, or replication after entry. For E-protein mutations in particular, defining the responsible stage will require direct comparison of peripheral viral kinetics, CNS entry, and post-entry brain replication.

Nonstructural protein variation should also be considered when interpreting TMUV CNS-associated phenotypes. Evidence from other flaviviruses suggests different functional roles for these proteins: NS1-family proteins can affect endothelial barrier function (76), whereas NS3 and NS5 primarily influence replication and antiviral immune responses (75, 90). Direct evidence linking these mechanisms to TMUV CNS disease, however, remains limited. Changes in replication or innate immune antagonism could modify systemic viral burden or post-entry CNS replication without directly altering the route of CNS access. NS protein-mediated IFN antagonism is therefore better considered a potential modifier of TMUV replication and inflammatory responses than an established mechanism of BBB dysfunction.

The priority for TMUV is therefore to move from sequence–phenotype associations to causal genotype–phenotype relationships. Reverse-genetic analysis of candidate mutations, combined with time-resolved measurements of peripheral replication, CNS entry, brain replication, BBB integrity, and neuropathology, will be needed to determine which stage is altered. Current evidence more directly implicates E-protein variation in CNS-associated phenotypes, whereas the contribution of nonstructural proteins remains less clearly defined.

### 5.4 Host inflammatory responses and BBB dysfunction after TMUV CNS entry

Host inflammatory responses and BBB dysfunction represent a second layer of TMUV neuropathogenesis after CNS access has occurred. The strongest TMUV-specific evidence for this temporal relationship comes from a duckling infection study showing that CNS entry can precede overt BBB disruption. In that model, viral particles were detected in brain tissue at an early stage when obvious clinical signs and increased BBB permeability were not yet apparent. Neurological signs, marked BBB disruption, and inflammatory cytokine accumulation appeared later (27). These observations indicate that detectable brain infection preceded overt barrier breakdown in this model, supporting secondary BBB injury after CNS infection was established.

The duckling study therefore separates early CNS infection from later BBB disruption. Barrier injury became evident only after local inflammatory responses had developed. Cytokines, chemokines, MMP activity, leukocyte recruitment, and glial activation can then act on the neurovascular unit and further weaken barrier integrity. Increased permeability may in turn facilitate additional immune-cell entry and exposure of CNS tissues to circulating inflammatory mediators, thereby amplifying neurological injury (91).

Whether this sequence is shared across TMUV strains and hosts remains unknown. The available evidence derives from a specific duckling model, whereas emerging or variant strains with stronger neurological phenotypes may differ in the timing of viremia, CNS entry, brain replication, inflammatory activation, and BBB injury. Defining these temporal differences will be important for determining whether barrier dysfunction is consistently a post-entry event or shifts earlier in infection in some TMUV backgrounds.

More severe neurological disease therefore cannot be attributed to BBB injury alone. It may result from earlier CNS access, greater post-entry replication, broader CNS-associated cell susceptibility, stronger inflammation, or more extensive barrier damage. If BBB injury appears only after brain infection and inflammatory activation, it is better interpreted as a downstream injury event or contributor to disease progression. If permeability increases before or at the earliest detectable stage of brain viral accumulation, it would support a more direct role in CNS access (6). These possibilities can only be distinguished by aligning BBB measurements with viremia, CNS viral kinetics, anatomical distribution, inflammatory responses, and neurological outcome.

Glial activation is another unresolved component of TMUV-associated neurovascular injury. Once TMUV reaches the CNS, microglia and astrocytes could contribute to cytokine and chemokine production, endothelial activation, leukocyte recruitment, MMP induction, and secondary injury to the neurovascular unit. This mechanism offers one explanation for why BBB dysfunction may become more pronounced after encephalitic changes have begun. Whether glial activation actively drives this barrier injury or mainly accompanies established CNS inflammation remains unclear. Studies that track viral replication, glial responses, endothelial injury, and BBB leakage over the same time course will be needed to resolve this relationship.

Current TMUV evidence therefore supports at least one model in which CNS infection precedes overt BBB disruption. The main unresolved issue is whether this temporal relationship is conserved across strains and infection settings, or whether BBB involvement shifts earlier in disease in variants with stronger CNS-associated phenotypes. Resolving this will require time-resolved studies that distinguish CNS entry, post-entry replication, barrier injury, and neuroinflammatory amplification.

## 6 Future perspectives: defining the timing and function of BBB dysfunction in TMUV CNS disease

Future work on TMUV should place CNS findings within a defined experimental and temporal context. Strain background, host species, infection route, sampling time, and anatomical site all influence how brain infection and BBB changes are interpreted. Mechanisms described for JEV, WNV, ZIKV, DENV, and TBEV provide useful comparisons, but their relevance to TMUV requires direct testing.

### 6.1 Defining the timing and route of TMUV CNS entry

A major priority is to establish when TMUV first reaches the CNS and whether measurable BBB changes occur at that stage. Peripheral infection models are particularly informative because they preserve the sequence from systemic replication and viremia to CNS exposure and brain infection. Serial sampling during early infection should therefore combine CNS viral detection with measurements of BBB permeability, endothelial activation, and junctional integrity. This would allow early barrier changes to be distinguished from disruption that develops only after virus is already present in the brain.

The location of the earliest viral signal is equally informative. Perivascular or microvascular localization would favor a vascular route, whereas early detection in the choroid plexus or cerebrospinal fluid would implicate the blood–cerebrospinal fluid interface. Initial involvement of the olfactory epithelium or olfactory bulb would support an olfactory route, while early viral detection in peripheral nerves or the brainstem would suggest neural spread. Combining these spatial data with BBB measurements should help define how TMUV enters the CNS rather than simply documenting that brain infection has occurred.

### 6.2 Testing BBB injury and neuroinflammatory amplification

The available duckling data provide a starting point for testing whether BBB injury follows early TMUV CNS infection. The next step is to determine whether the same temporal relationship is observed in other viral lineages, hosts, and infection settings, particularly in strains with stronger CNS-associated phenotypes.

Direct comparison of classical and emerging TMUV strains under matched conditions would be especially informative. Standardizing inoculation route, dose, host age, and early sampling intervals would allow differences in CNS viral kinetics and BBB injury to be compared without major experimental confounding. The critical measurements are the onset of CNS infection, subsequent brain viral expansion, and the timing and severity of barrier disruption. A strain may therefore produce greater BBB injury because it reaches the CNS earlier, replicates more efficiently after entry, or induces stronger local inflammation, even when barrier breakdown itself is not required for neuroinvasion.

Resolving this sequence requires parallel analysis of CNS infection and neurovascular injury. Viral localization and cell-type identification can define the onset and distribution of CNS infection, while BBB permeability, junctional integrity, endothelial responses, MMP activity, and glial activation can establish when barrier damage develops. If these changes consistently follow detectable CNS infection, they would support secondary BBB injury.

The next question is whether this later barrier damage contributes actively to disease progression. This can be addressed by relating the magnitude of BBB leakage to leukocyte recruitment, glial activation, neuronal injury, and neurological severity after CNS infection is established. A close temporal association between worsening barrier dysfunction and increasing neuroinflammatory injury would support an amplifying role, particularly if manipulation of barrier or inflammatory pathways modifies neurological outcome without altering the initial timing of CNS entry.

### 6.3 Linking TMUV genetic variation to specific stages of CNS disease and BBB dysfunction

Reverse genetics provides the most direct way to establish causal links between TMUV sequence variation and CNS-associated phenotypes. Candidate mutations identified from classical and emerging strains should be introduced individually or in defined combinations and tested under matched infection conditions. Particular attention should be given to E-protein substitutions already associated with altered CNS phenotypes, while candidate nonstructural changes can be evaluated for effects on replication and host responses.

The key experimental distinction is between CNS access and events that occur after entry. Peripheral infection can reveal changes in viremia, timing of CNS detection, and BBB involvement, whereas direct CNS inoculation bypasses the entry step and tests replication and disease within the CNS itself. A mutation that alters disease only after peripheral infection would implicate systemic dissemination or CNS access; persistence of the phenotype after direct CNS inoculation would instead support an effect on post-entry replication or neurovirulence. BBB-relevant cell systems can further determine whether the same mutation directly alters endothelial infection, permeability, or inflammatory responses.

Combining these approaches would allow individual TMUV mutations to be assigned to specific stages of disease rather than simply classified as virulence determinants.

### 6.4 Developing BBB-relevant models and CNS readouts for TMUV research

BBB-relevant models should be selected according to the specific stage of TMUV CNS disease being investigated. Endothelial monolayer and Transwell systems are useful for examining direct interactions between TMUV and brain endothelial cells, including barrier crossing, permeability changes, TEER reduction, junctional disruption, and endothelial inflammatory responses (39). They can also distinguish direct viral effects from changes induced by viral proteins, infected-cell supernatants, or inflammatory mediators. However, endothelial monocultures cannot fully reproduce the neurovascular unit, and co-culture or multicellular systems incorporating astrocytes, pericytes, microglia, or choroid plexus-associated cells are better suited to studying barrier regulation and neuroinflammatory crosstalk (92).

Species-relevant *in vitro* and *ex vivo* systems are especially important because TMUV is primarily an avian flavivirus. Duck- or goose-derived brain endothelial cells, glial cells, choroid plexus epithelial cells, brain slices, and organoid-like systems would provide a more relevant setting for testing TMUV–host interactions. These models could be used to compare endothelial susceptibility, barrier crossing, replication in CNS-associated cells, and inflammatory responses among TMUV strains. Mammalian systems remain useful for comparative work, but they cannot be assumed to reproduce avian neurovascular biology.

Avian infection models are essential for studying the complete course of TMUV neuroinvasion in the natural host. Duck and goose models retain the progression from peripheral replication and viremia to CNS entry, brain infection, BBB changes, and neurological disease. They are therefore particularly valuable for comparing classical and emerging strains and for determining whether stronger CNS phenotypes reflect earlier CNS access, greater systemic viral burden, broader brain dissemination, or more severe post-entry injury.

Mouse models address a different part of the disease process. Several studies show strong route-dependent susceptibility, with robust disease after direct CNS inoculation but limited or inconsistent infection after peripheral routes (86). Peripheral mouse infection may therefore be less informative for natural neuroinvasion, whereas direct CNS inoculation can be used to examine replication, encephalitic inflammation, neural injury, and BBB changes once the entry step has been bypassed. This makes direct-CNS mouse models particularly useful for assessing CNS-intrinsic neurovirulence.

The value of each model therefore depends on the disease step being tested. *In vitro* BBB systems are best suited to endothelial interaction and barrier function, avian systems to host-relevant neuroinvasion, and direct CNS models to post-entry replication and injury. Readouts should be chosen accordingly rather than applied as a uniform panel across all models. This stage-matched strategy would provide a clearer basis for separating CNS access, secondary BBB damage, neuroinflammatory amplification, and CNS-intrinsic disease severity in TMUV infection.

### 6.5 Integrating CNS access, BBB dysfunction, and neuropathogenesis in TMUV infection

A useful next step is to reconstruct TMUV CNS disease as a temporal sequence from CNS access to post-entry replication, barrier injury, neuroinflammation, and neurological damage. Viral determinants and host responses can then be placed within this sequence according to the stage they alter. Figure 3 summarizes this integrated framework.

**Figure 3:**
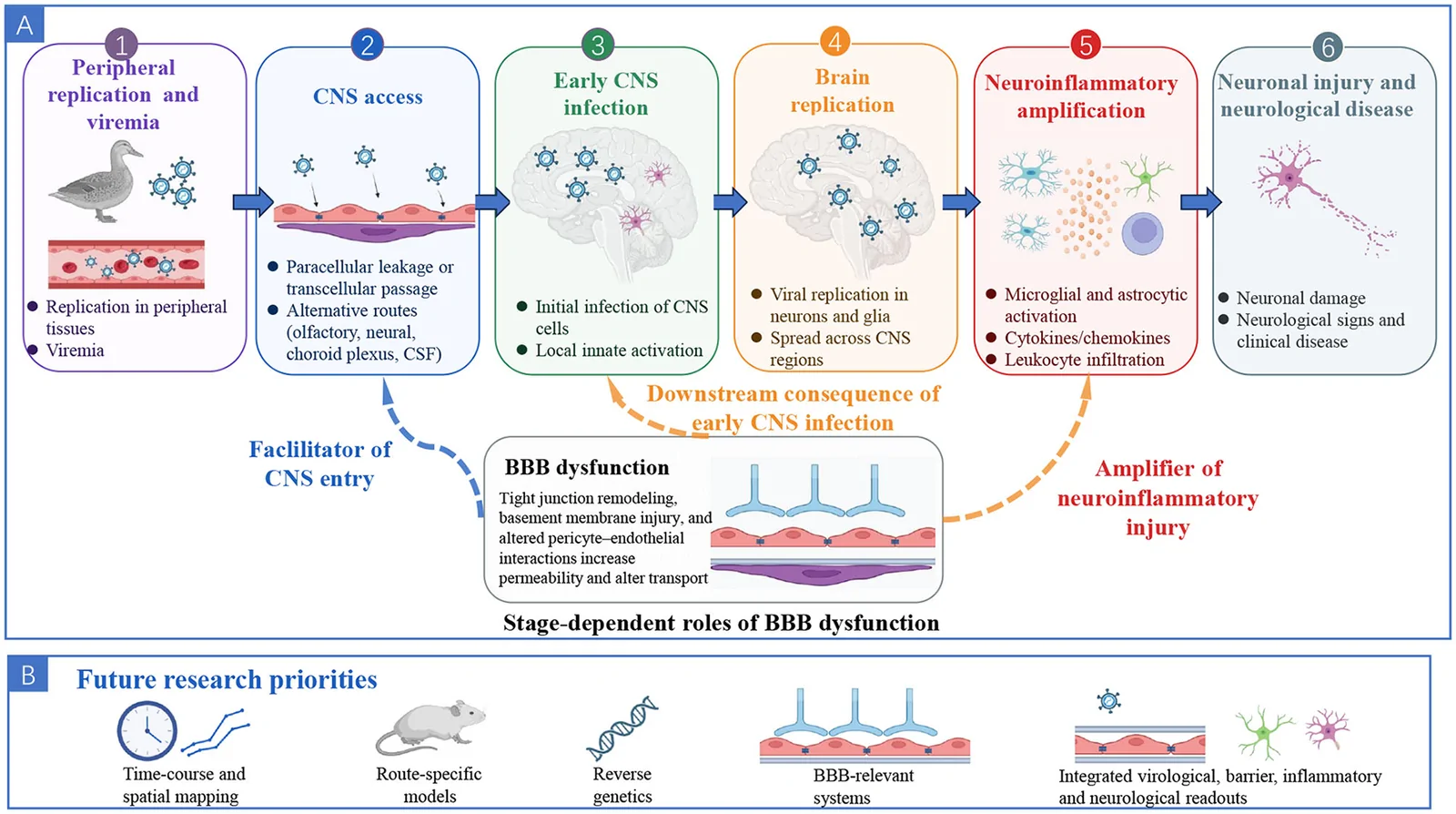
Stage-specific framework for TMUV neuroinvasion, BBB dysfunction, and neuropathogenesis. **(A)** TMUV-associated CNS disease is organized as a peripheral-to-CNS sequence linking peripheral replication and viremia, CNS access, early CNS infection, brain replication, neuroinflammatory amplification, and neuronal injury and neurological disease. BBB dysfunction is not treated as a single mechanism of viral entry; its role depends on timing and disease context. It may facilitate CNS access, emerge after early CNS infection as secondary barrier injury, or intensify neuroinflammatory damage after brain infection has been established. **(B)** Future research priorities for assigning BBB dysfunction and viral determinants to defined pathogenic stages, including time-course and spatial mapping, route-specific infection models, reverse genetics, BBB-relevant systems, and integrated virological, barrier, inflammatory, and neurological readouts.

Together, natural avian host models, direct CNS inoculation models, BBB-relevant systems, and reverse genetics can assign viral and host factors to the disease step they affect. This will strengthen interpretation of TMUV neuroinvasion and neuropathogenesis and provide a clearer basis for evaluating strain virulence, viral attenuation, vaccine safety, and disease control strategies.

## 7 Conclusions

This review brings together evidence on BBB dysfunction in neurotropic flavivirus infection and applies it to a stage-specific interpretation of TMUV neuroinvasion and neuropathogenesis. Studies of JEV, WNV, ZIKV, DENV, and TBEV show that flavivirus-associated CNS disease proceeds through a multi-step peripheral-to-CNS sequence rather than a single event. Across these viruses, BBB dysfunction is a recurrent component of CNS disease, but its timing, mechanism, and relative contribution differ substantially according to virus species, host, route of infection, sampling time, and experimental model.

For TMUV, available studies now support CNS involvement through neurological signs, brain lesions, viral detection in CNS tissues, and strain-dependent CNS-associated phenotypes. These observations are important, but they should be treated as evidence of CNS involvement rather than as direct evidence of a defined CNS entry route, BBB-dependent neuroinvasion, or CNS-intrinsic neurovirulence. Stronger CNS-associated phenotypes in emerging or variant TMUV strains may reflect changes in systemic replication, CNS access, CNS-associated cell susceptibility, post-entry brain replication, BBB dysfunction, or neuroinflammation. The biological meaning of these phenotypes therefore depends on the stage of infection at which the underlying change occurs.

A central challenge for TMUV research is to resolve the sequence linking peripheral infection, CNS entry, brain replication, BBB injury, and neuroinflammation. Temporal and anatomical analysis will be needed to determine whether stronger CNS-associated phenotypes arise from more efficient entry, greater post-entry replication, or secondary neurovascular injury. Such distinctions are essential for TMUV strain assessment and vaccine safety evaluation, because virulence and attenuation cannot be judged reliably from endpoint brain viral load, neurological signs, BBB leakage, or mortality alone.

## References

[1] Pierson TC Diamond MS. The continued threat of emerging flaviviruses. Nat Microbiol. (2020) 56:796–812. doi: 10.1038/s41564-020-0714-0 32367055 PMC7696730

[2] Sips GJ Wilschut J Smit JM. Neuroinvasive flavivirus infections. Rev Med Virol. (2012) 222:69–87. doi: 10.1002/rmv.712 22086854

[3] Cody SG Adam A Siniavin A Kang SS Wang T. Flaviviruses-induced neurological sequelae. Pathogens. (2024) 14:22. doi: 10.3390/pathogens14010022 39860983 PMC11768111

[4] Chen Z Li G. Immune response and blood-brain barrier dysfunction during viral neuroinvasion. Innate Immun. (2021) 272:109–17. doi: 10.1177/1753425920954281 32903111 PMC7882805

[5] Galea I. The blood-brain barrier in systemic infection and inflammation. Cell Mol Immunol. (2021) 1811:2489–501. doi: 10.1038/s41423-021-00757-x 34594000 PMC8481764

[6] Mustafá YM Meuren LM Coelho SVA de Arruda LB. Pathways exploited by flaviviruses to counteract the blood-brain barrier and invade the central nervous system. Front Microbiol. (2019) 10:525. doi: 10.3389/fmicb.2019.00525 30984122 PMC6447710

[7] Verma S Lo Y Chapagain M Lum S Kumar M Gurjav U . West Nile virus infection modulates human brain microvascular endothelial cells tight junction proteins and cell adhesion molecules: transmigration across the *in vitro* blood-brain barrier. Virology. (2009) 3852:425–33. doi: 10.1016/j.virol.2008.11.047 19135695 PMC2684466

[8] Puerta-Guardo H Glasner DR Espinosa DA Biering SB Patana M Ratnasiri K . Flavivirus NS1 triggers tissue-specific vascular endothelial dysfunction reflecting disease tropism. Cell Rep. (2019) 266:1598–613.e8. doi: 10.1016/j.celrep.2019.01.036 30726741 PMC6934102

[9] Cui Y Pan Y Guo J Wang D Tong X Wang Y . The evolution, genomic epidemiology, and transmission dynamics of tembusu virus. Viruses. (2022) 14:1236. doi: 10.3390/v14061236 35746707 PMC9227414

[10] Yu G Lin Y Tang Y Diao Y. Evolution of Tembusu Virus in Ducks, Chickens, Geese, Sparrows, and Mosquitoes in Northern China. Viruses. (2018) 10:485. doi: 10.3390/v10090485 30201873 PMC6164154

[11] Zhu Y Hu Z Lv X Huang R Gu X Zhang C. et al. A novel Tembusu virus isolated from goslings in China form a new subgenotype 211. Transbound Emerg Dis. (2022) 694:1782–93. doi: 10.1111/tbed.14155 33993639

[12] Yang Q Ding Y Yao W Chen S Jiang Y Yang L . Pathogenicity and Interspecies Transmission of Cluster 3 Tembusu Virus Strain TMUV HQ-22 Isolated from Geese. Viruses. (2023) 15:2449. doi: 10.3390/v15122449 38140690 PMC10747935

[13] Misra UK Kalita J. Overview: Japanese encephalitis. Prog Neurobiol. (2010) 912:108–20. doi: 10.1016/j.pneurobio.2010.01.008 20132860

[14] Sejvar JJ. Clinical manifestations and outcomes of West Nile virus infection. Viruses. (2014) 62:606–23. doi: 10.3390/v6020606 24509812 PMC3939474

[15] Bogovic P Strle F. Tick-borne encephalitis: a review of epidemiology, clinical characteristics, and management. World J Clin Cases. (2015) 35:430–41. doi: 10.12998/wjcc.v3.i5.430 25984517 PMC4419106

[16] Ming GL Tang H Song H. Advances in zika virus research: stem cell models, challenges, and opportunities. Cell Stem Cell. (2016) 196:690–702. doi: 10.1016/j.stem.2016.11.014 27912090 PMC5218815

[17] Carod-Artal FJ Wichmann O Farrar J Gascón J. Neurological complications of dengue virus infection. Lancet Neurol. (2013) 129:906–19. doi: 10.1016/S1474-4422(13)70150-9 23948177

[18] de Vries L Harding AT. Mechanisms of neuroinvasion and neuropathogenesis by pathologic flaviviruses. Viruses. (2023) 15:261. doi: 10.3390/v15020261 36851477 PMC9965671

[19] Su J Li S Hu X Yu X Wang Y Liu P . Duck egg-drop syndrome caused by BYD virus, a new Tembusu-related flavivirus. PLoS ONE. (2011) 63:e18106. doi: 10.1371/journal.pone.0018106 21455312 PMC3063797

[20] Yan P Zhao Y Zhang X Xu D Dai X Teng Q . An infectious disease of ducks caused by a newly emerged Tembusu virus strain in mainland China. Virology. (2011) 4171:1–8. doi: 10.1016/j.virol.2011.06.003 21722935

[21] Lei W Guo X Fu S Feng Y Tao X Gao X . The genetic characteristics and evolution of Tembusu virus. Vet Microbiol. (2017) 201:32–41. doi: 10.1016/j.vetmic.2017.01.003 28284620

[22] Fang Y Hang T Yang LM Xue JB Fujita R Feng XS . Long-distance spread of Tembusu virus, and its dispersal in local mosquitoes and domestic poultry in Chongming Island, China. Infect Dis Poverty. (2023) 121:52. doi: 10.1186/s40249-023-01098-9 37218001 PMC10200701

[23] Tang Y Diao Y Yu C Gao X Ju X Xue C . Characterization of a Tembusu virus isolated from naturally infected house sparrows (Passer domesticus) in Northern China. Transbound Emerg Dis. (2013) 602:152–8. doi: 10.1111/j.1865-1682.2012.01328.x 22515847

[24] Tang Y Diao Y Chen H Ou Q Liu X Gao X . Isolation and genetic characterization of a tembusu virus strain isolated from mosquitoes in Shandong, China. Transbound Emerg Dis. (2015) 622:209–16. doi: 10.1111/tbed.12111 23711093

[25] Hu D Wu C Wang R Yao X Nie K Lv Q . Persistence of tembusu virus in culex tritaeniorhynchus in Yunnan Province, China. Pathogens. (2023) 12:490. doi: 10.3390/pathogens12030490 36986412 PMC10058924

[26] Huang Y Hu Z Dong J Li L Zhang J Kuang R. et al. Chicken-origin Cluster 32 Tembusu virus exhibits higher infectivity than duck-origin Cluster 2 Tembusu virus in chicks. Front Vet Sci. (2023) 10:1152802. doi: 10.3389/fvets.2023.1152802 37035806 PMC10080150

[27] Yang S Huang Y Shi Y Bai X Yang P Chen Q. Tembusu Virus entering the central nervous system caused nonsuppurative encephalitis without disrupting the blood-brain barrier. J Virol. (2021) 95:e02191–20. doi: 10.1128/JVI.02191-20 33472933 PMC8092698

[28] Yang S Shi Y Wu J Chen Q. Ultrastructural study of the duck brain infected with duck Tembusu virus. Front Microbiol. (2023) 14:1086828. doi: 10.3389/fmicb.2023.1086828 36891400 PMC9987711

[29] Yan D Shi Y Wang H Li G Li X Wang B . A Single Mutation at Position 156 in the Envelope Protein of Tembusu Virus Is Responsible for Virus Tissue Tropism and Transmissibility in Ducks. J Virol. (2018) 92:e00427–18. doi: 10.1128/JVI.00427-18 29899104 PMC6096821

[30] Zhang W Jiang B Zeng M Duan Y Wu Z Wu Y . Binding of Duck Tembusu Virus Nonstructural Protein 2A to Duck STING Disrupts Induction of Its Signal Transduction Cascade To Inhibit Beta Interferon Induction. J Virol. (2020) 94:e01850-19. doi: 10.1128/JVI.01850-19 32075929 PMC7163140

[31] Marshall EM Koopmans MPG Rockx B. A Journey to the Central Nervous System: Routes of Flaviviral Neuroinvasion in Human Disease. Viruses. (2022) 14:2096. doi: 10.3390/v14102096 36298652 PMC9611789

[32] Suen WW Prow NA Hall RA Bielefeldt-Ohmann H. Mechanism of West Nile virus neuroinvasion: a critical appraisal. Viruses. (2014) 67:2796–825. doi: 10.3390/v6072796 25046180 PMC4113794

[33] Maximova OA Pletnev AG. Flaviviruses and the Central Nervous System: Revisiting Neuropathological Concepts. Annu Rev Virol. (2018) 51:255–72. doi: 10.1146/annurev-virology-092917-043439 30265628

[34] May Fulton C Bailey WJ. Live viral vaccine neurovirulence screening: current and future models. Vaccines. (2021) 9:710. doi: 10.3390/vaccines9070710 34209433 PMC8310194

[35] Profaci CP Munji RN Pulido RS Daneman R. The blood-brain barrier in health and disease: Important unanswered questions. J Exp Med. (2020) 21:62. doi: 10.1084/jem.20190062 32211826 PMC7144528

[36] Lochhead JJ Yang J Ronaldson PT Davis TP. Structure, function, and regulation of the blood-brain barrier tight junction in central nervous system disorders. Front Physiol. (2020) 11:914. doi: 10.3389/fphys.2020.00914 32848858 PMC7424030

[37] Castro Dias M Mapunda JA Vladymyrov M Engelhardt B. Structure and junctional complexes of endothelial, epithelial and glial brain barriers. Int J Mol Sci. (2019) 20:5372. doi: 10.3390/ijms20215372 31671721 PMC6862204

[38] Saunders NR Dziegielewska KM Møllgård K Habgood MD. Markers for blood-brain barrier integrity: how appropriate is Evans blue in the twenty-first century and what are the alternatives? Front Neurosci. (2015) 9:385. doi: 10.3389/fnins.2015.00385 26578854 PMC4624851

[39] Helms HC Abbott NJ Burek M Cecchelli R Couraud PO Deli MA . *In vitro* models of the blood-brain barrier: an overview of commonly used brain endothelial cell culture models and guidelines for their use. J Cereb Blood Flow Metab. (2016) 365:862–90. doi: 10.1177/0271678X16630991 26868179 PMC4853841

[40] Ahishali B Kaya M. Evaluation of blood-brain barrier integrity using vascular permeability markers: evans blue, sodium fluorescein, albumin-alexa fluor conjugates, and horseradish peroxidase. Methods Mol Biol. (2021) 2367:87–103. doi: 10.1007/7651_2020_316 32785841

[41] Patabendige A Michael BD Craig AG Solomon T. Brain microvascular endothelial-astrocyte cell responses following Japanese encephalitis virus infection in an *in vitro* human blood-brain barrier model. Mol Cell Neurosci. (2018) 89:60–70. doi: 10.1016/j.mcn.2018.04.002 29635016 PMC5984247

[42] Daniels BP Holman DW Cruz-Orengo L Jujjavarapu H Durrant DM Klein RS. Viral pathogen-associated molecular patterns regulate blood-brain barrier integrity via competing innate cytokine signals. MBio. (2014) 55:e01476–14. doi: 10.1128/mBio.01476-14 25161189 PMC4173776

[43] Li F Wang Y Yu L Cao S Wang K Yuan J . Viral infection of the central nervous system and neuroinflammation precede blood-brain barrier disruption during japanese encephalitis virus infection. J Virol. (2015) 8910:5602–14. doi: 10.1128/JVI.00143-15 25762733 PMC4442524

[44] Bardina SV Michlmayr D Hoffman KW Obara CJ Sum J Charo IF . Differential roles of chemokines CCL2 and CCL7 in monocytosis and leukocyte migration during west nile virus infection. J Immunol. (2015) 1959:4306–18. doi: 10.4049/jimmunol.1500352 26401006 PMC4610864

[45] Wang K Wang H Lou W Ma L Li Y Zhang N . IP-10 promotes blood-brain barrier damage by inducing tumor necrosis factor alpha production in japanese encephalitis. Front Immunol. (2018) 9:1148. doi: 10.3389/fimmu.2018.01148 29910805 PMC5992377

[46] Roe K Kumar M Lum S Orillo B Nerurkar VR Verma S. West Nile virus-induced disruption of the blood-brain barrier in mice is characterized by the degradation of the junctional complex proteins and increase in multiple matrix metalloproteinases. J Gen Virol. (2012) 93Pt 6:1193–203. doi: 10.1099/vir.0.040899-0 22398316 PMC3755517

[47] Alimonti JB Ribecco-Lutkiewicz M Sodja C Jezierski A Stanimirovic DB Liu Q . Zika virus crosses an *in vitro* human blood brain barrier model. Fluids Barriers CNS. (2018) 151:15. doi: 10.1186/s12987-018-0100-y 29759080 PMC5952854

[48] Hsieh JT St John AL. Japanese encephalitis virus and its mechanisms of neuroinvasion. PLoS Pathog. (2020) 164:e1008260. doi: 10.1371/journal.ppat.1008260 32240272 PMC7117652

[49] Rempe RG Hartz AMS Bauer B. Matrix metalloproteinases in the brain and blood-brain barrier: Versatile breakers and makers. J Cereb Blood Flow Metab. (2016) 369:1481–507. doi: 10.1177/0271678X16655551 27323783 PMC5012524

[50] Wang P Dai J Bai F Kong KF Wong SJ Montgomery RR . Matrix metalloproteinase 9 facilitates West Nile virus entry into the brain. J Virol. (2008) 8218:8978–85. doi: 10.1128/JVI.00314-08 18632868 PMC2546894

[51] Ashley SL Pretto CD Stier MT Kadiyala P Castro-Jorge L Hsu TH . Matrix Metalloproteinase Activity in Infections by an Encephalitic Virus, Mouse Adenovirus Type 1. J Virol. (2017) 916. doi: 10.1128/JVI.01412-16 28053109 PMC5331797

[52] Verma S Kumar M Gurjav U Lum S Nerurkar VR. Reversal of West Nile virus-induced blood-brain barrier disruption and tight junction proteins degradation by matrix metalloproteinases inhibitor. Virology. (2010) 3971:130–8. doi: 10.1016/j.virol.2009.10.036 19922973 PMC3102050

[53] Chen CJ Ou YC Li JR Chang CY Pan HC Lai CY . Infection of pericytes *in vitro* by Japanese encephalitis virus disrupts the integrity of the endothelial barrier. J Virol. (2014) 882:1150–61. doi: 10.1128/JVI.02738-13 24198423 PMC3911661

[54] Chang CY Li JR Chen WY Ou YC Lai CY Hu YH . Disruption of *in vitro* endothelial barrier integrity by Japanese encephalitis virus-Infected astrocytes. Glia. (2015) 6311:1915–32. doi: 10.1002/glia.22857 25959931

[55] Pan P Li G Shen M Yu Z Ge W Lao Z . DENV NS1 and MMP-9 cooperate to induce vascular leakage by altering endothelial cell adhesion and tight junction. PLoS Pathog. (2021) 177:e1008603. doi: 10.1371/journal.ppat.1008603 34310658 PMC8341711

[56] Papa MP Meuren LM Coelho SVA Lucas CGO Mustafá YM Lemos Matassoli F . Zika virus infects, activates, and crosses brain microvascular endothelial cells, without barrier disruption. Front Microbiol. (2017) 8:2557. doi: 10.3389/fmicb.2017.02557 29312238 PMC5743735

[57] Marchetti L Engelhardt B. Immune cell trafficking across the blood-brain barrier in the absence and presence of neuroinflammation. Vasc Biol. (2020) 21:H1–h18. doi: 10.1530/VB-19-0033 32923970 PMC7439848

[58] Roe K Orillo B Verma S. West Nile virus-induced cell adhesion molecules on human brain microvascular endothelial cells regulate leukocyte adhesion and modulate permeability of the *in vitro* blood-brain barrier model. PLoS ONE. (2014) 97:e102598. doi: 10.1371/journal.pone.0102598 25036379 PMC4103843

[59] Glass WG Lim JK Cholera R Pletnev AG Gao JL Murphy PM. Chemokine receptor CCR5 promotes leukocyte trafficking to the brain and survival in West Nile virus infection. J Exp Med. (2005) 2028:1087–98. doi: 10.1084/jem.20042530 16230476 PMC2213214

[60] Paul AM Acharya D Duty L Thompson EA Le L Stokic DS . Osteopontin facilitates West Nile virus neuroinvasion via neutrophil “Trojan horse” transport. Sci Rep. (2017) 71:4722. doi: 10.1038/s41598-017-04839-7 28680095 PMC5498593

[61] Lannes N Summerfield A Filgueira L. Regulation of inflammation in Japanese encephalitis. J Neuroinflammation. (2017) 141:158. doi: 10.1186/s12974-017-0931-5 28807053 PMC5557552

[62] Malavige GN Ogg GS. Pathogenesis of vascular leak in dengue virus infection. Immunology. (2017) 1513:261–9. doi: 10.1111/imm.12748 28437586 PMC5461104

[63] Potokar M Jorgačevski J Zorec R. Astrocytes in flavivirus infections. Int J Mol Sci. (2019) 20:691. doi: 10.3390/ijms20030691 30736273 PMC6386967

[64] Smit JM Moesker B Rodenhuis-Zybert I Wilschut J. Flavivirus cell entry and membrane fusion. Viruses. (2011) 32:160–71. doi: 10.3390/v3020160 22049308 PMC3206597

[65] Zhang X Jia R Shen H Wang M Yin Z Cheng A. Structures and functions of the envelope glycoprotein in flavivirus infections. Viruses. (2017) 91:338. doi: 10.3390/v9110338 29137162 PMC5707545

[66] Zhang S Li L Woodson SE Huang CY Kinney RM Barrett AD . A mutation in the envelope protein fusion loop attenuates mouse neuroinvasiveness of the NY99 strain of West Nile virus. Virology. (2006) 3531:35–40. doi: 10.1016/j.virol.2006.05.025 16806383

[67] Kobayashi S Kaneko C Kawakami R Hasebe R Sawa H Yoshii K . Amino acid 159 of the envelope protein affects viral replication and T-cell infiltration by West Nile virus in intracranial infection. Sci Rep. (2020) 101:7168. doi: 10.1038/s41598-020-64199-7 32346055 PMC7189269

[68] Yang J Yang H Li Z Wang W Lin H Liu L . Envelope protein mutations L107F and E138K are important for neurovirulence attenuation for japanese encephalitis virus SA14-14-2 strain. Viruses. (2017) 9:20. doi: 10.3390/v9010020 28117725 PMC5294989

[69] Huang R He Y Zhang C Luo Y Chen C Tan N . The mutation of Japanese encephalitis virus envelope protein residue 389 attenuates viral neuroinvasiveness. Virol J. (2024) 211:128. doi: 10.1186/s12985-024-02398-8 38840203 PMC11151615

[70] Shan C Xia H Haller SL Azar SR Liu Y Liu J . A Zika virus envelope mutation preceding the 2015 epidemic enhances virulence and fitness for transmission. Proc Natl Acad Sci U S A. (2020) 11733:20190–7. doi: 10.1073/pnas.2005722117 32747564 PMC7443865

[71] Liu Z Zhang Y Cheng M Ge N Shu J Xu Z . A single nonsynonymous mutation on ZIKV E protein-coding sequences leads to markedly increased neurovirulence *in vivo*. Virol Sin. (2022) 371:115–26. doi: 10.1016/j.virs.2022.01.021 35234632 PMC8922429

[72] Carbaugh DL Baric RS Lazear HM. Envelope protein glycosylation mediates zika virus pathogenesis. J Virol. (2019) 93:e00113–19. doi: 10.1128/JVI.00113-19 30944176 PMC6613755

[73] Fishburn AT Pham OH Kenaston MW Beesabathuni NS Shah PS. Let's get physical: flavivirus-host protein-protein interactions in replication and pathogenesis. Front Microbiol. (2022) 13:847588. doi: 10.3389/fmicb.2022.847588 35308381 PMC8928165

[74] Goh JZH De Hayr L Khromykh AA Slonchak A. The flavivirus non-structural protein 5 (NS5): structure, functions, and targeting for development of vaccines and therapeutics. Vaccines. (2024) 12:865. doi: 10.3390/vaccines12080865 39203991 PMC11360482

[75] Chen S Wu Z Wang M Cheng A. Innate immune evasion mediated by flaviviridae non-structural proteins. Viruses. (2017) 9:291. doi: 10.3390/v9100291 28991176 PMC5691642

[76] Zeng Q Liu J Hao C Zhang B Zhang H. Making sense of flavivirus non-strctural protein 1 in innate immune evasion and inducing tissue-specific damage. Virus Res. (2023) 336:199222. doi: 10.1016/j.virusres.2023.199222 37716670 PMC10518729

[77] Puerta-Guardo H Glasner DR Harris E. Dengue virus NS1 disrupts the endothelial glycocalyx, leading to hyperpermeability. PLoS Pathog. (2016) 127:e1005738. doi: 10.1371/journal.ppat.1005738 27416066 PMC4944995

[78] Glasner DR Ratnasiri K Puerta-Guardo H Espinosa DA Beatty PR Harris E. Dengue virus NS1 cytokine-independent vascular leak is dependent on endothelial glycocalyx components. PLoS Pathog. (2017) 1311:e1006673. doi: 10.1371/journal.ppat.1006673 29121099 PMC5679539

[79] Wessel AW Dowd KA Biering SB Zhang P Edeling MA Nelson CA . Levels of circulating NS1 impact west nile virus spread to the brain. J Virol. (2021) 9520:e0084421. doi: 10.1128/JVI.00844-21 34346770 PMC8475509

[80] Zhang L Nan X Zhou D Wang X Zhu S Li Q . Japanese encephalitis virus NS1 and N1′ protein disrupts the blood-brain barrier through macrophage migration inhibitory factor-mediated autophagy. J Virol. (2024) 985:e0011624. doi: 10.1128/jvi.00116-24 PMC11092347 38591880

[81] Lin RJ Chang BL Yu HP Liao CL Lin YL. Blocking of interferon-induced Jak-Stat signaling by Japanese encephalitis virus NS5 through a protein tyrosine phosphatase-mediated mechanism. J Virol. (2006) 8012:5908–18. doi: 10.1128/JVI.02714-05 16731929 PMC1472572

[82] Ye J Chen Z Li Y Zhao Z He W Zohaib A . Japanese encephalitis virus NS5 inhibits type i interferon (IFN) production by blocking the nuclear translocation of IFN regulatory factor 3 and NF-κB. J Virol. (2017) 91:e00039–17. doi: 10.1128/JVI.00039-17 28179530 PMC5375679

[83] Hu T Wu Z Wu S Chen S Cheng A. The key amino acids of E protein involved in early flavivirus infection: viral entry. Virol J. (2021) 181:136. doi: 10.1186/s12985-021-01611-2 34217298 PMC8254458

[84] Li N Zhao J Yang Y Zeng Y Liu S. Innate immune responses to duck Tembusu virus infection. Vet Res. (2020) 511:87. doi: 10.1186/s13567-020-00814-9 32641107 PMC7346331

[85] Zhang W Zeng M Jiang B Lu T Guo J Hu T . Amelioration of beta interferon inhibition by ns4b contributes to attenuating tembusu virus virulence in ducks. Front Immunol. (2021) 12:671471. doi: 10.3389/fimmu.2021.671471 34079553 PMC8165282

[86] Li S Li X Zhang L Wang Y Yu X Tian K . Duck Tembusu virus exhibits neurovirulence in BALB/c mice. Virol J. (2013) 10:260. doi: 10.1186/1743-422X-10-260 23941427 PMC3765187

[87] Li Q Huang J Hu X Peng D Song Y Zhang D. Pathogenicity of a chicken-origin cluster 3 isolate of Tembusu virus in Pekin ducklings and Kunming mice. Res Vet Sci. (2025) 190:105670. doi: 10.1016/j.rvsc.2025.105670 40311168

[88] Cheng Y Wang R Wu Q Chen J Wang A Wu Z . Advancements in research on duck tembusu virus infections. Viruses. (2024) 16:811. doi: 10.3390/v16050811 38793692 PMC11126125

[89] He Y Guo J Wang X Wu Z Wang T Wang M . The A487 residue in the E protein of duck Tembusu virus significantly enhances viral replication and increases its neurovirulence in Kunming mice. J Virol. (2025) 996:e0030825. doi: 10.1128/jvi.00308-25 40401981 PMC12172445

[90] van den Elsen K Quek JP Luo D. Molecular insights into the flavivirus replication complex. Viruses. (2021) 13:956. doi: 10.3390/v13060956 34064113 PMC8224304

[91] Takata F Nakagawa S Matsumoto J Dohgu S. Blood-brain barrier dysfunction amplifies the development of neuroinflammation: understanding of cellular events in brain microvascular endothelial cells for prevention and treatment of bbb dysfunction. Front Cell Neurosci. (2021) 15:661838. doi: 10.3389/fncel.2021.661838 34588955 PMC8475767

[92] Stone NL England TJ O'Sullivan SE. A novel transwell blood brain barrier model using primary human cells. Front Cell Neurosci. (2019) 13:230. doi: 10.3389/fncel.2019.00230 31244605 PMC6563620

